# Supplementary material for: The Cyclic Nitronate Route to Pharmaceutical Molecules: Synthesis of GSK’s Potent PDE4 Inhibitor as a Case Study
Source: Molecules. 2020 Aug 8;25(16):3613. doi: 10.3390/molecules25163613 (PMC7464803; doi:10.3390/molecules25163613)
Supplement: Supplementary file 1 [file molecules-25-03613-s001.pdf]

Supporting information

The Cyclic Nitronate Route to Pharmaceutical Molecules: Synthesis of GSK's Potent PDE4 Inhibitor as a Case Study

Evgeny V. Pospelov,[1,2] Ivan S. Golovanov,[1] Sema L. Ioffe,[1] Alexey Yu. Sukhorukov[1,3,*]

[1] N. D. Zelinsky Institute of Organic Chemistry, 119991, Leninsky prospect, 47, Moscow, Russia

[2] Department of Chemistry, M. V. Lomonosov Moscow State University, 119991, Leninskie gory, 1, str. 3, Moscow, Russia

[3] Plekhanov Russian University of Economics, 117997, Stremyanny per. 36, Moscow, Russia

* Correspondence: sukhorukov@ioc.ac.ru; Tel.: +7-499-135-53-29 (A.Yu.)

Contents

Copies of NMR spectra for products **10**, **17**, **18**, **18'**, **19**, **20** and **CMPO** S2

Copies of HPLC chromatograms for **CMPO** S42

DFT calculations S44

References S55

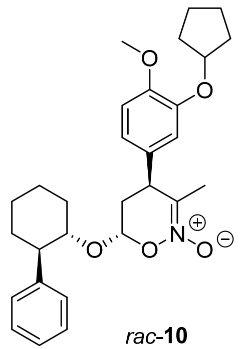

rac-**10**

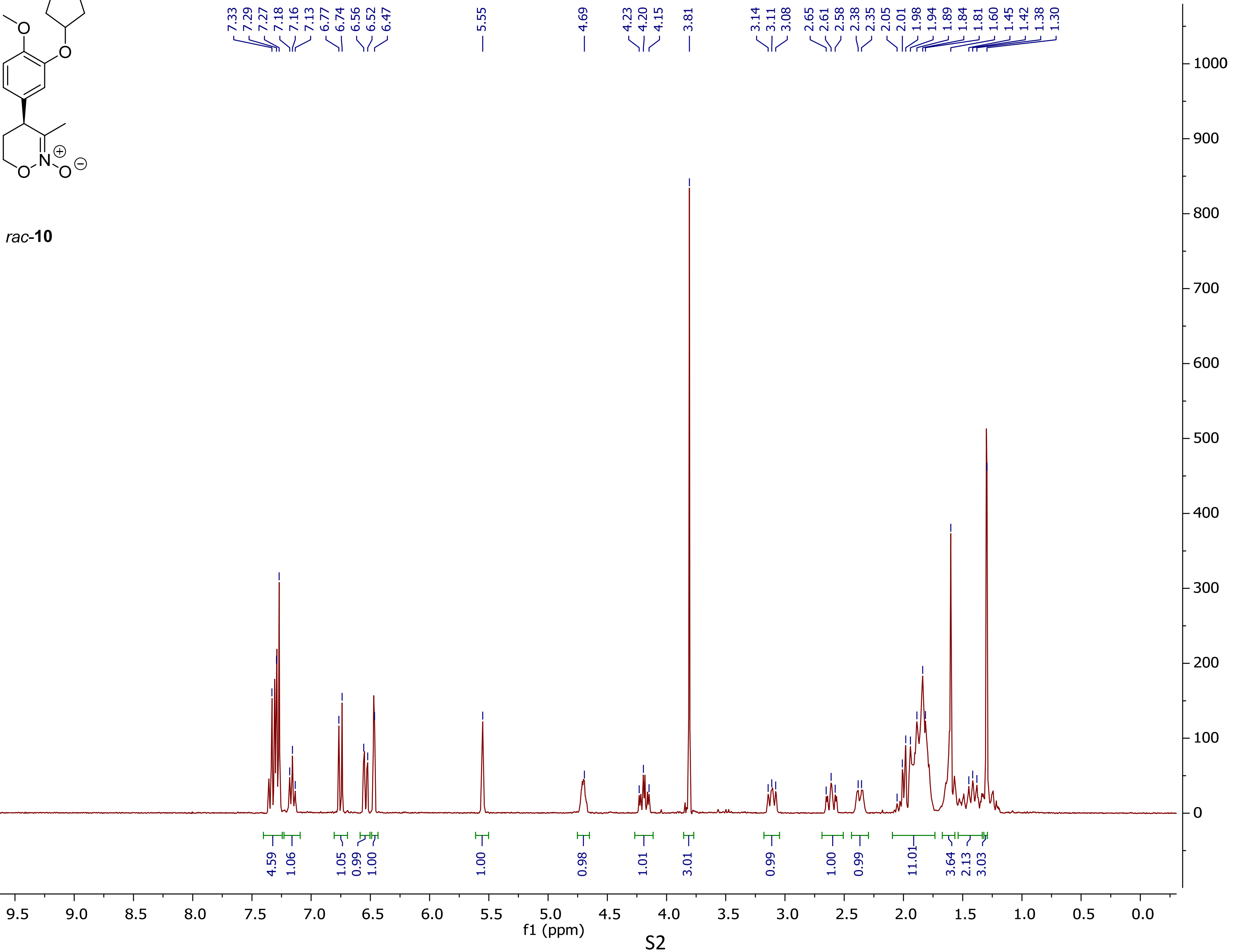
7.33
7.29
7.27
7.18
7.16
7.13
6.77
6.74
6.56
6.52
6.47
5.55
4.69
4.23
4.20
4.15
3.81
3.14
3.11
3.08
2.65
2.61
2.58
2.38
2.35
2.05
2.01
1.98
1.94
1.89
1.84
1.81
1.60
1.45
1.42
1.38
1.30
4.59
1.06
1.05
0.99
1.00
1.00
0.98
1.01
3.01
0.99
1.00
0.99
11.01
3.64
2.13
3.03
1000
900
800
700
600
500
400
300
200
100
0
10.0
9.5
9.0
8.5
8.0
7.5
7.0
6.5
6.0
5.5
5.0
4.5
4.0
3.5
3.0
2.5
2.0
1.5
1.0
0.5
0.0
f1 (ppm)

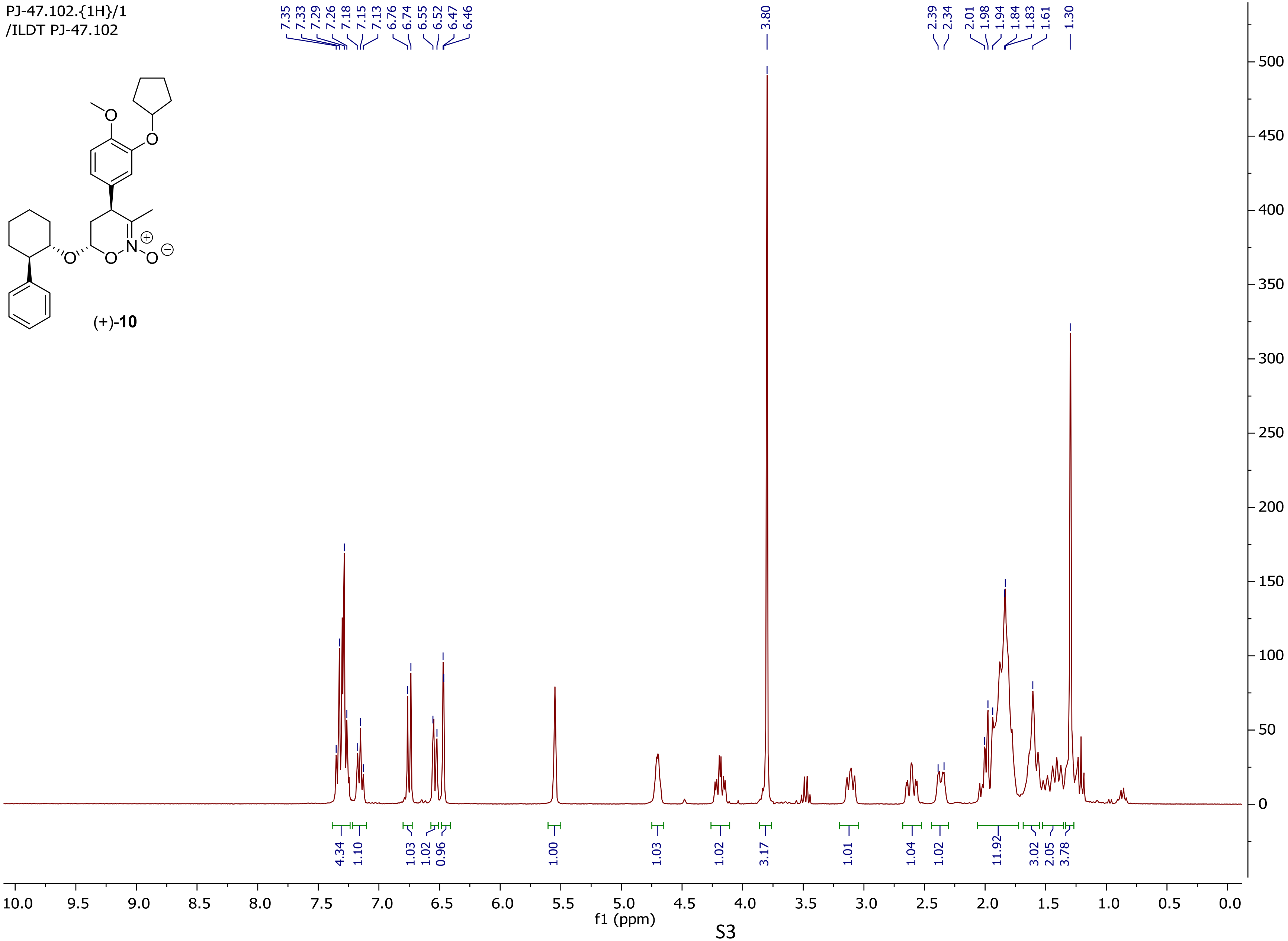
PJ-47.102.{1H}/1
/ILDT PJ-47.102
(+)-10
7.35 7.33 7.29 7.26 7.18 7.15 7.13 6.76 6.74 6.55 6.52 6.47 6.46
3.80
2.39 2.34 2.01 1.98 1.94 1.84 1.83 1.61 1.30
4.34 1.10 1.03 1.02 0.96 1.00 1.03 1.02 3.17 1.01 1.04 1.02 11.92 3.02 2.05 3.78
f1 (ppm)

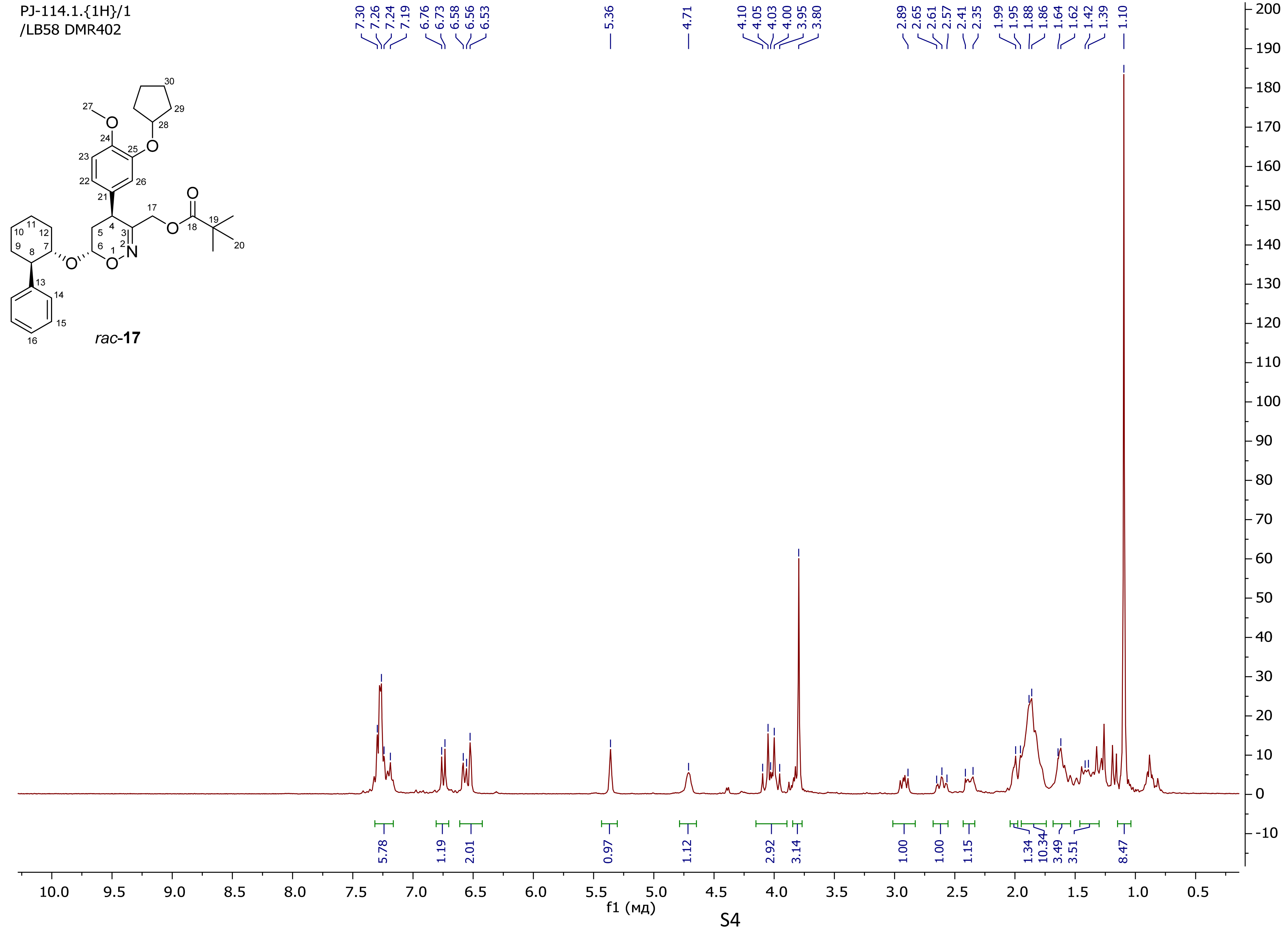
PJ-114.1.{1H}/1
/LB58 DMR402
7.30 7.26 7.24 7.19 6.76 6.73 6.58 6.56 6.53 5.36 4.71 4.10 4.05 4.03 4.00 3.95 3.80 2.89 2.65 2.61 2.57 2.41 2.35 1.99 1.95 1.88 1.86 1.64 1.62 1.42 1.39 1.10
5.78 1.19 2.01 0.97 1.12 2.92 3.14 1.00 1.00 1.15 1.34 10.34 3.49 3.51 8.47
f1 (мд)

rac-**17**

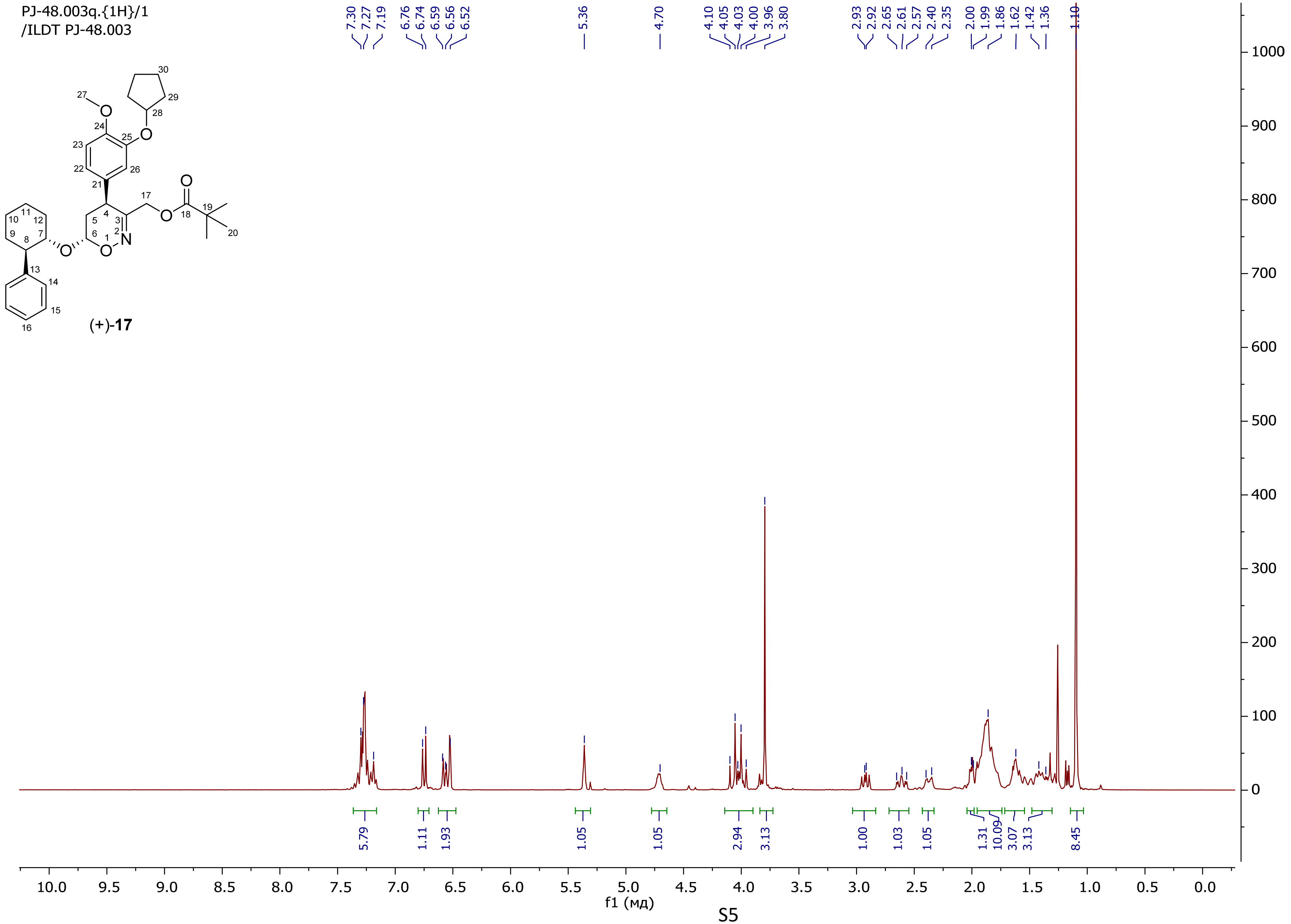
PJ-48.003q.{1H}/1
/ILDT PJ-48.003
7.30 7.27 7.19 6.76 6.74 6.59 6.56 6.52 5.36 4.70 4.10 4.05 4.03 4.00 3.96 3.80 2.93 2.92 2.65 2.61 2.57 2.40 2.35 2.00 1.99 1.86 1.62 1.42 1.36 1.10
(+)-17
5.79 1.11 1.93 1.05 1.05 2.94 3.13 1.00 1.03 1.05 1.31 10.09 3.07 3.13 8.45
f1 (мд)

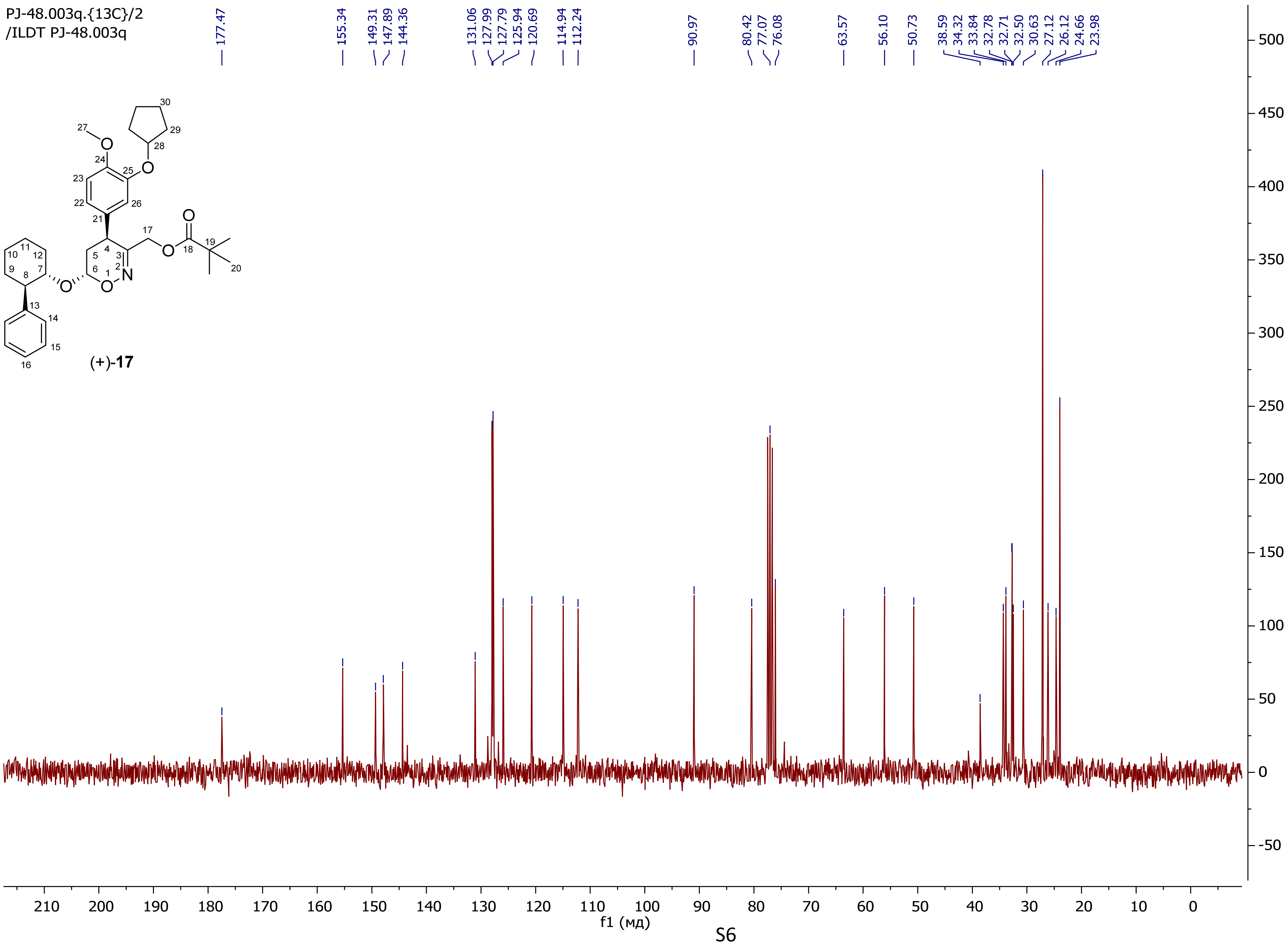
PJ-48.003q.{13C}/2
/ILDT PJ-48.003q
177.47
155.34
149.31
147.89
144.36
131.06
127.99
127.79
125.94
120.69
114.94
112.24
90.97
80.42
77.07
76.08
63.57
56.10
50.73
38.59
34.32
33.84
32.78
32.71
32.50
30.63
27.12
26.12
24.66
23.98
(+)-17
f1 (мд)

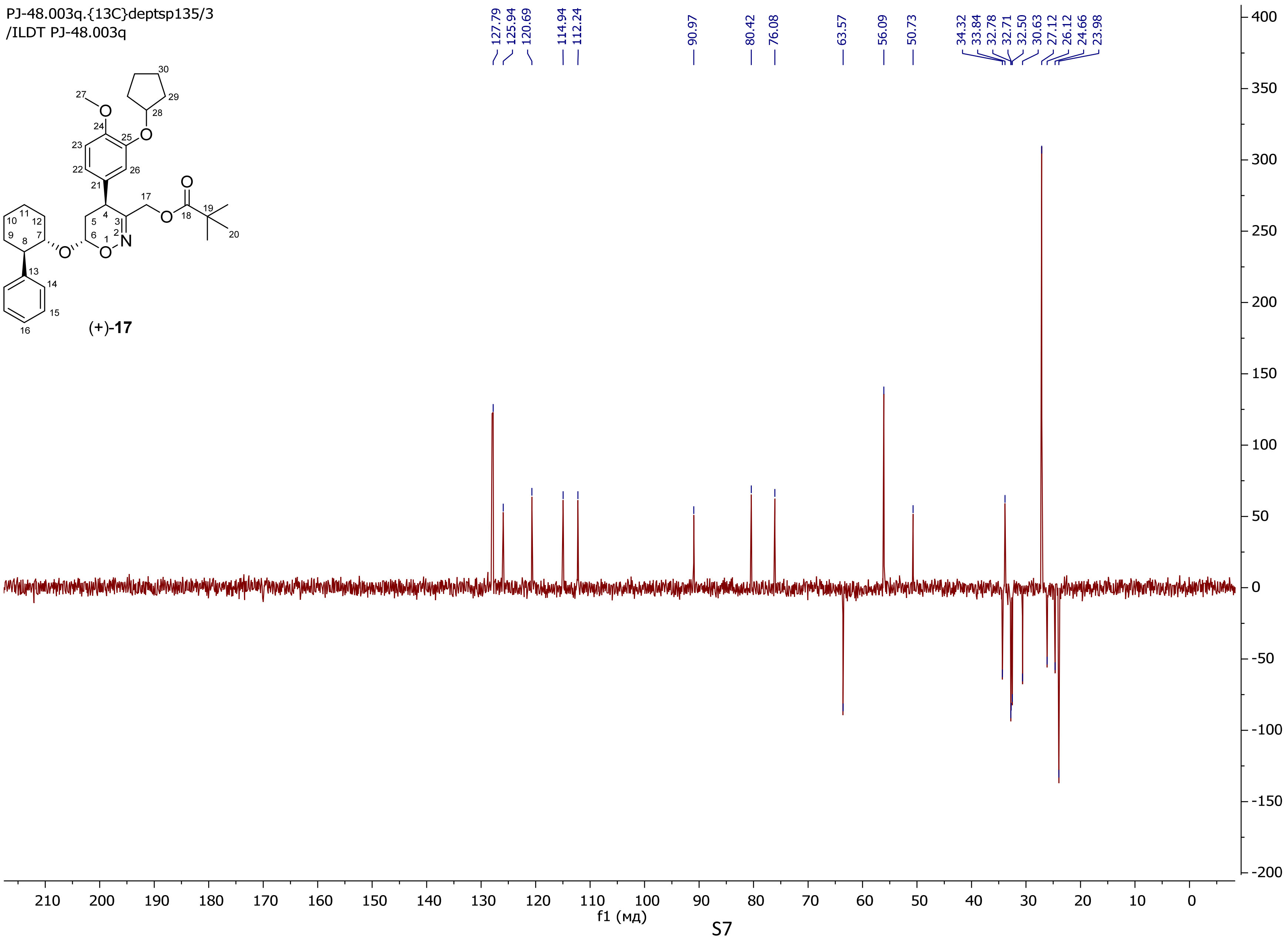
PJ-48.003q.{13C}deptsp135/3
/ILDT PJ-48.003q
(+)-17
127.79
125.94
120.69
114.94
112.24
90.97
80.42
76.08
63.57
56.09
50.73
34.32
33.84
32.78
32.71
32.50
30.63
27.12
26.12
24.66
23.98
f1 (мд)

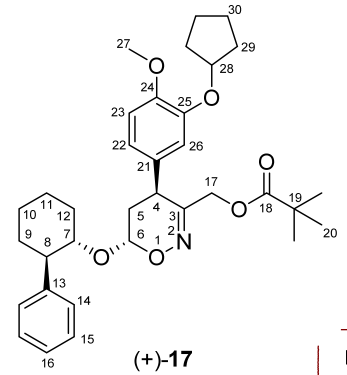

(+)-**17**

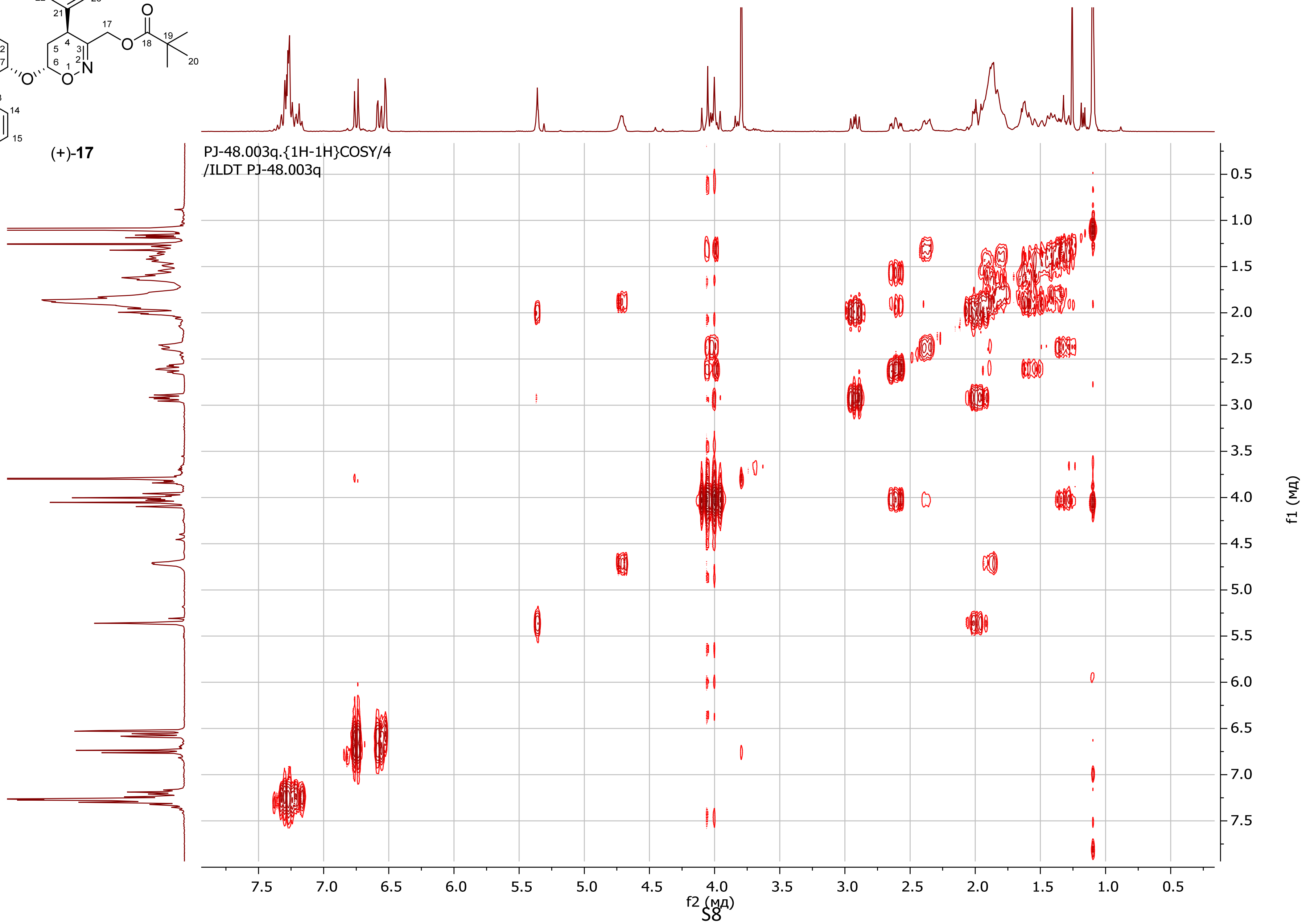

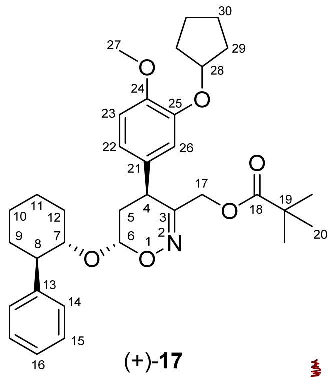

(+)-**17**

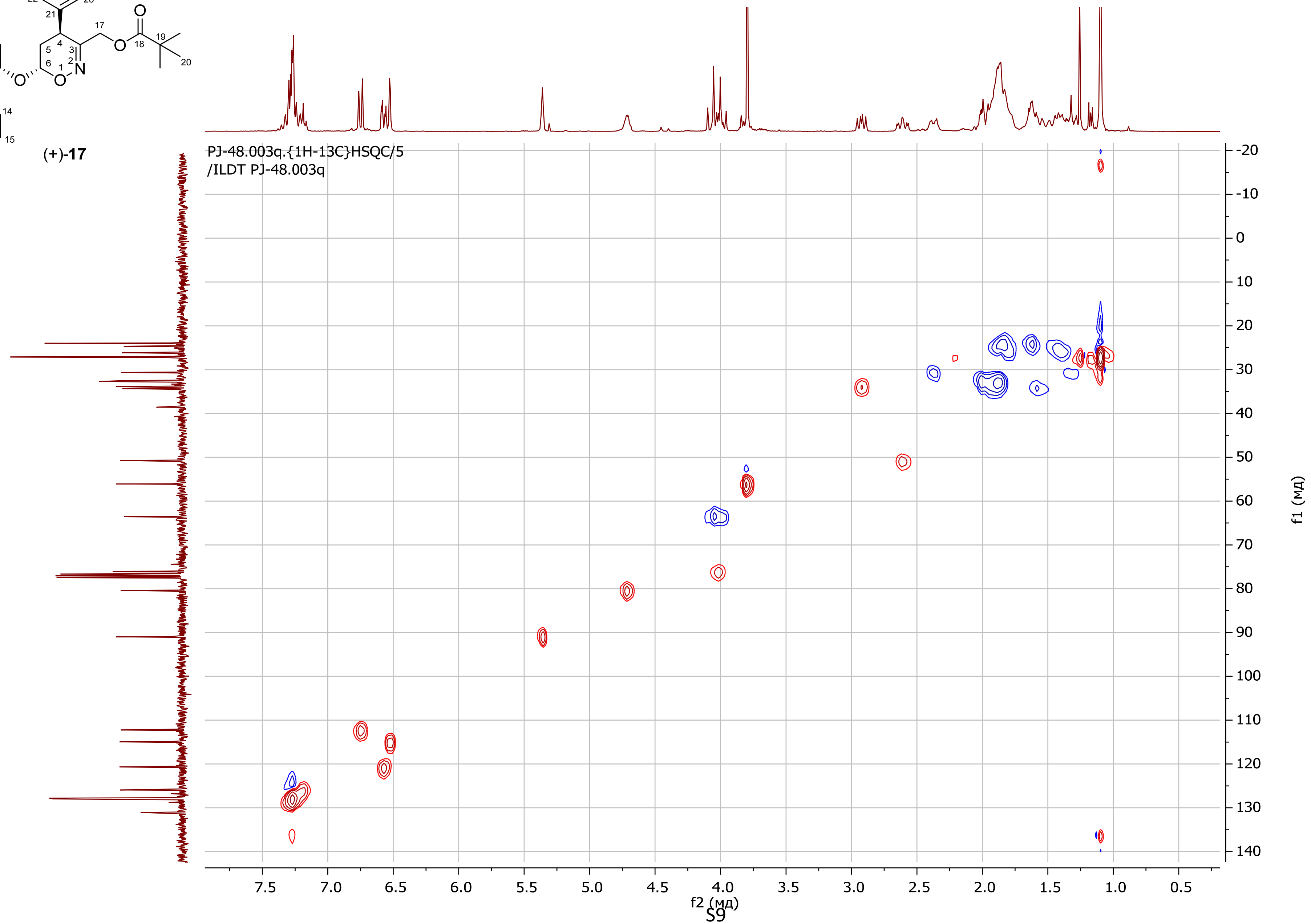

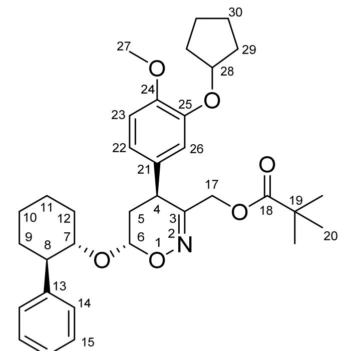

(+)-**17**

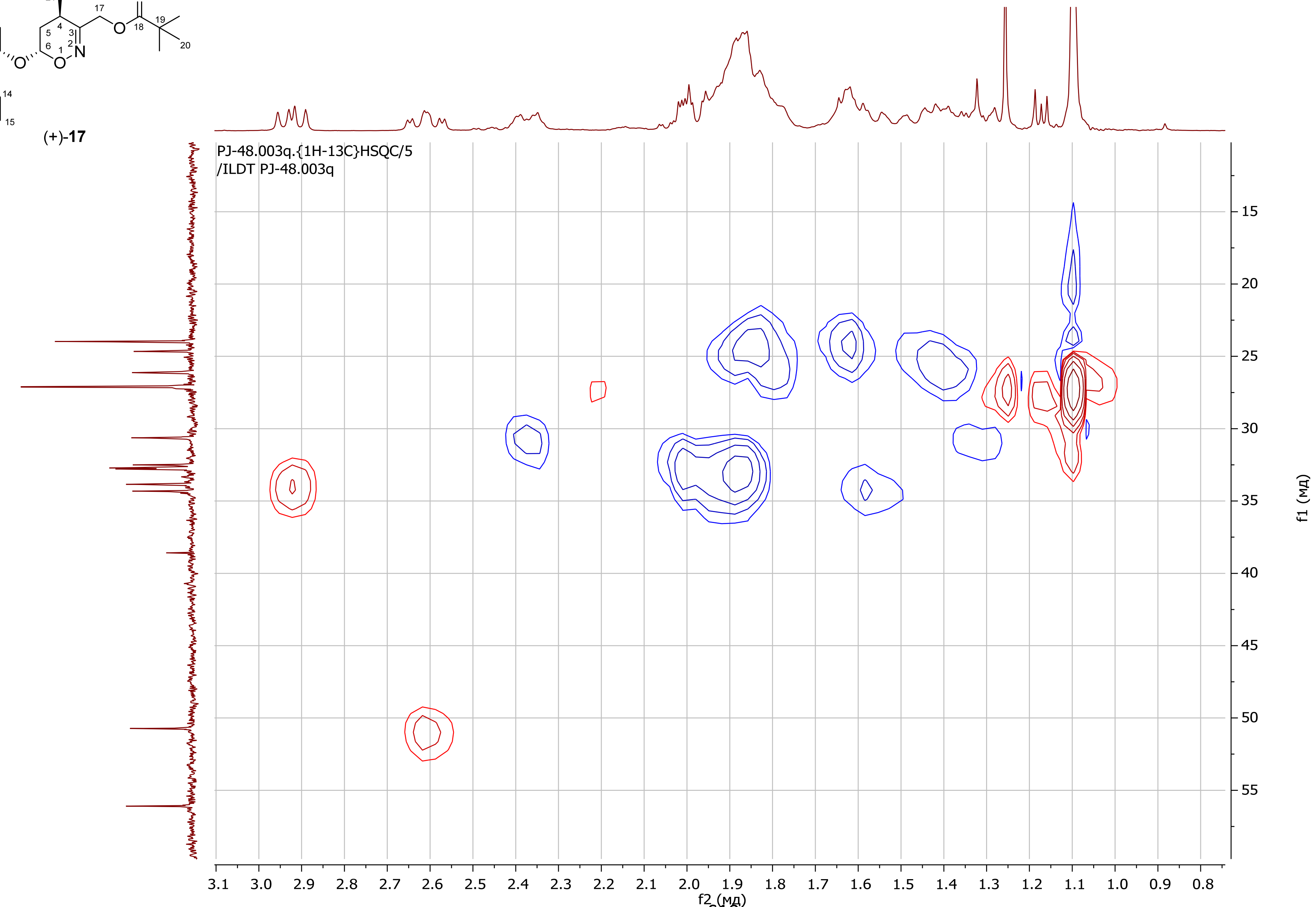

PJ-116.1.{1H}/1
/TERN i6514

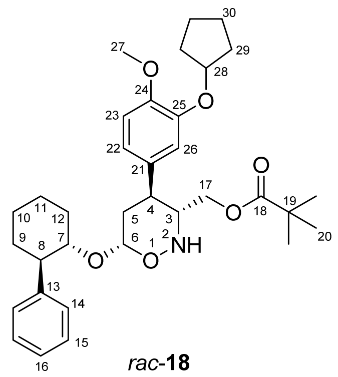

rac-**18**

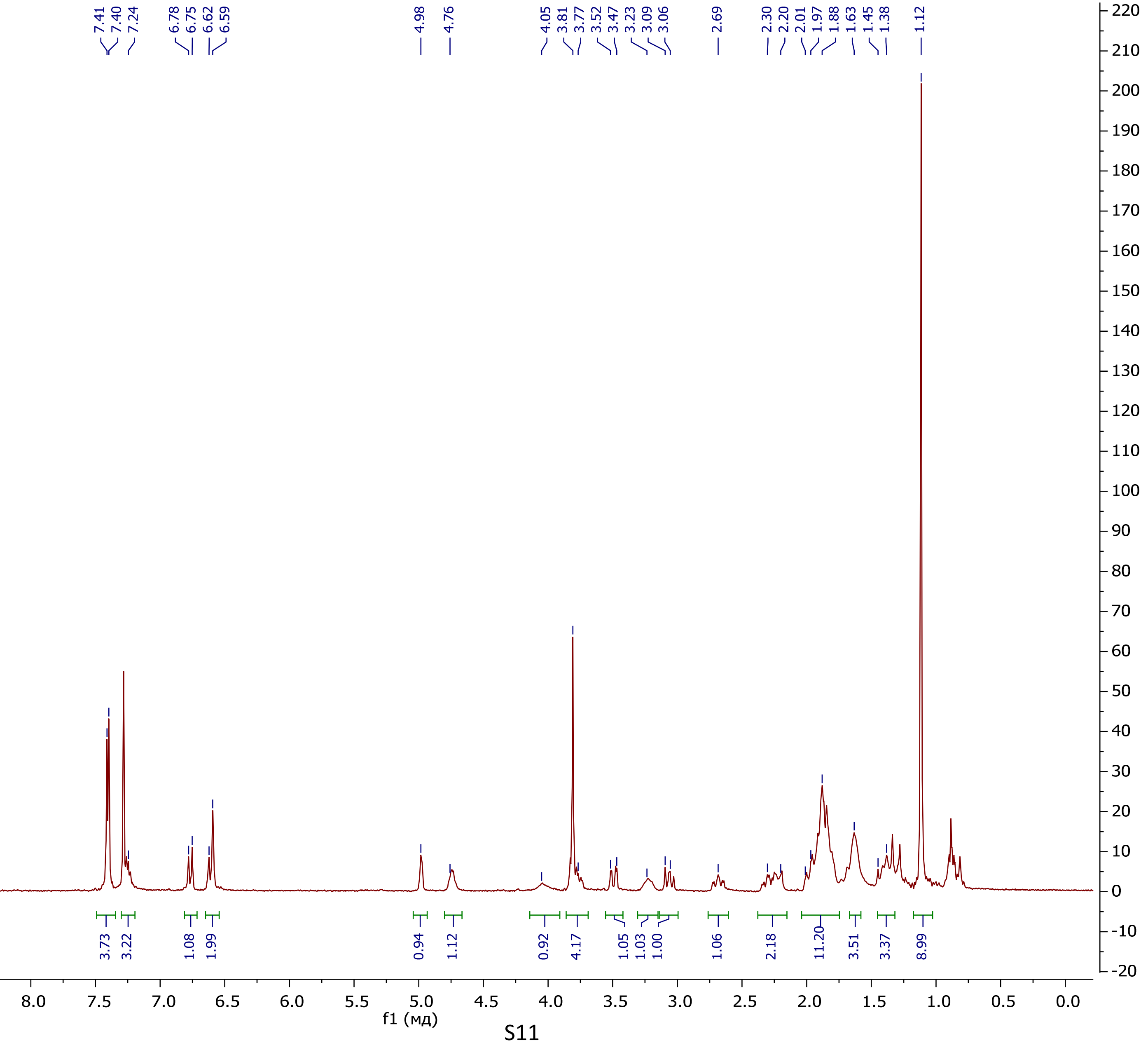

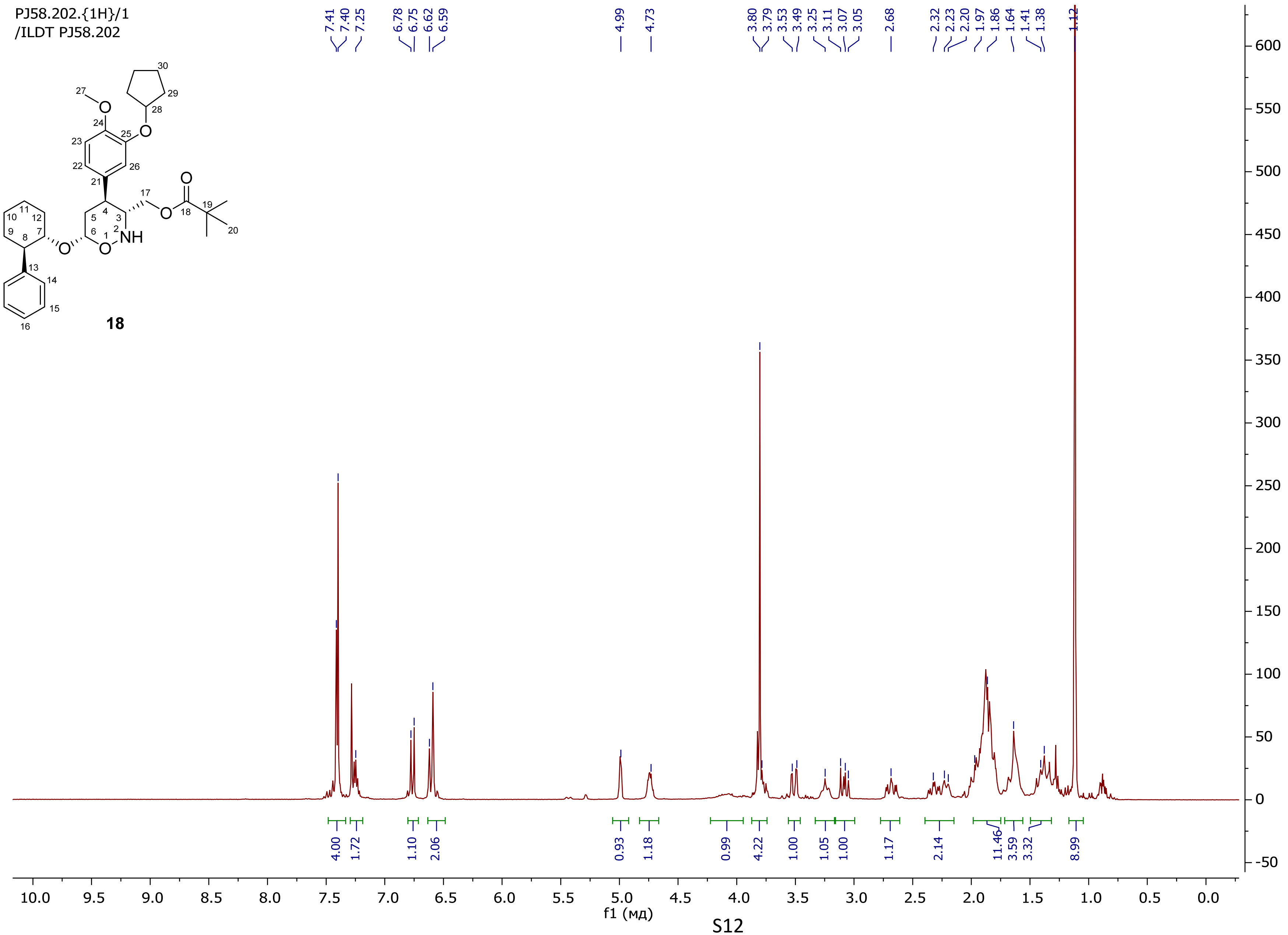
PJ58.202.{1H}/1
/ILDT PJ58.202
18
7.41
7.40
7.25
6.78
6.75
6.62
6.59
4.99
4.73
3.80
3.79
3.53
3.49
3.25
3.11
3.07
3.05
2.68
2.32
2.23
2.20
1.97
1.86
1.64
1.41
1.38
1.12
4.00
1.72
1.10
2.06
0.93
1.18
0.99
4.22
1.00
1.05
1.00
1.17
2.14
11.46
3.59
3.32
8.99
f1 (мд)

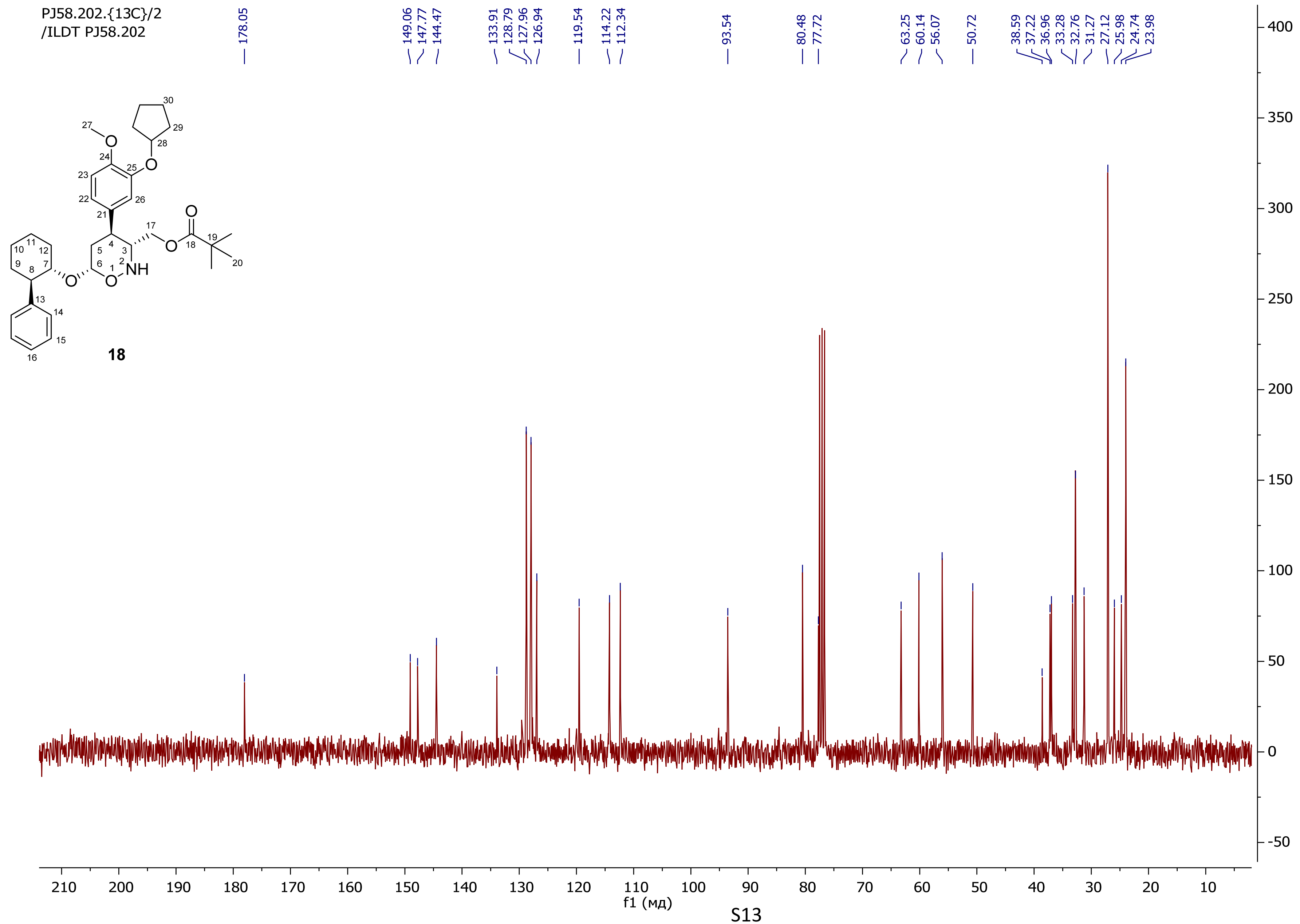
PJ58.202.{13C}/2
/ILDT PJ58.202
178.05
149.06
147.77
144.47
133.91
128.79
127.96
126.94
119.54
114.22
112.34
93.54
80.48
77.72
63.25
60.14
56.07
50.72
38.59
37.22
36.96
33.28
32.76
31.27
27.12
25.98
24.74
23.98
18
f1 (мд)

PJ58.202.{13C}deptsp135/3
/ILDT PJ58.202

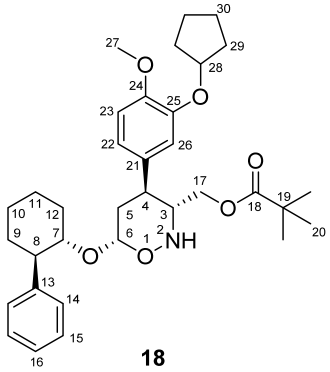

18

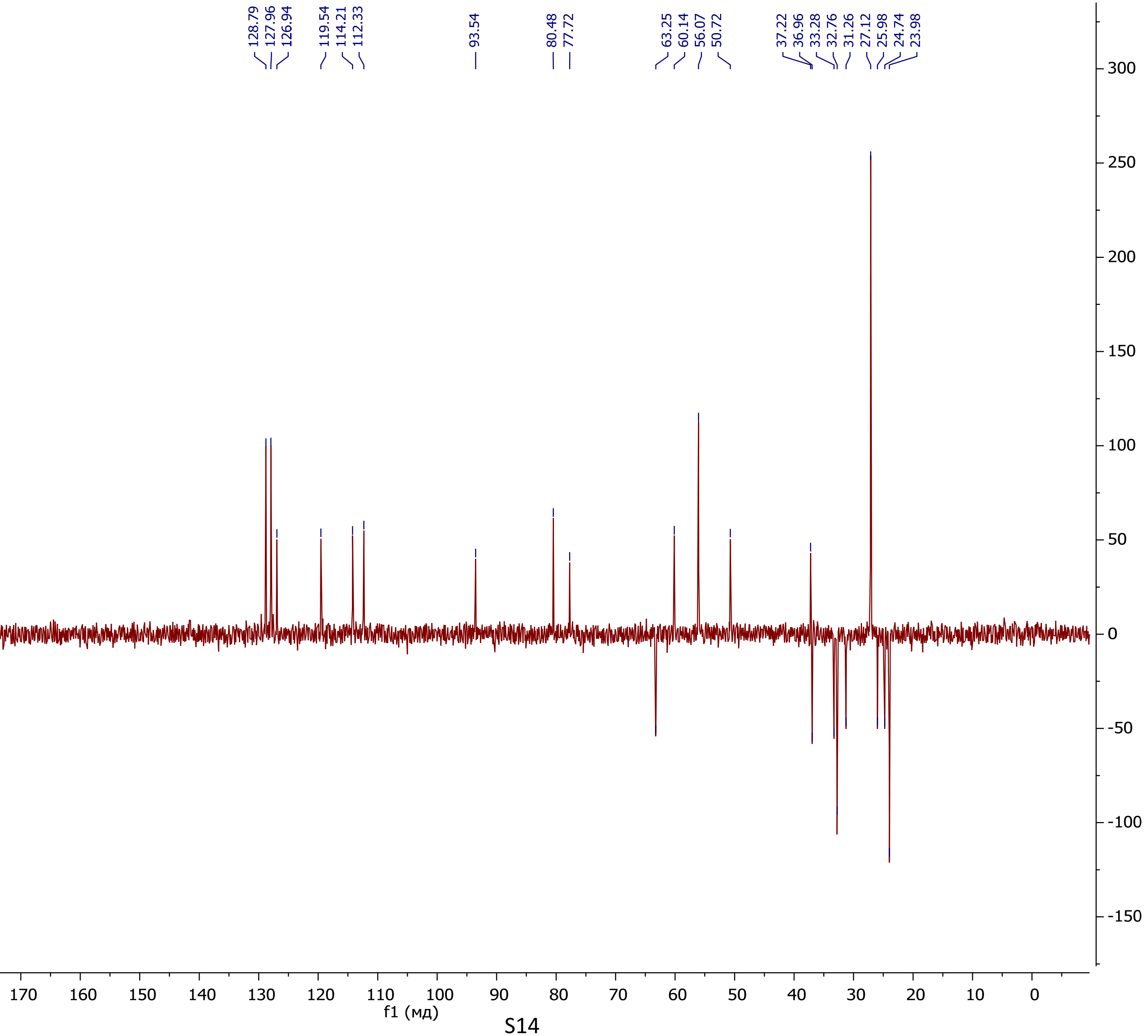

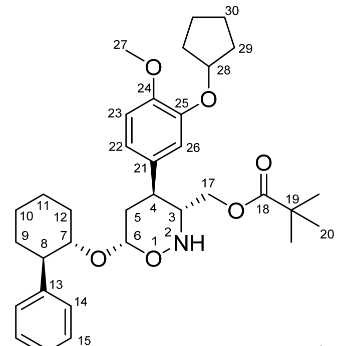

18

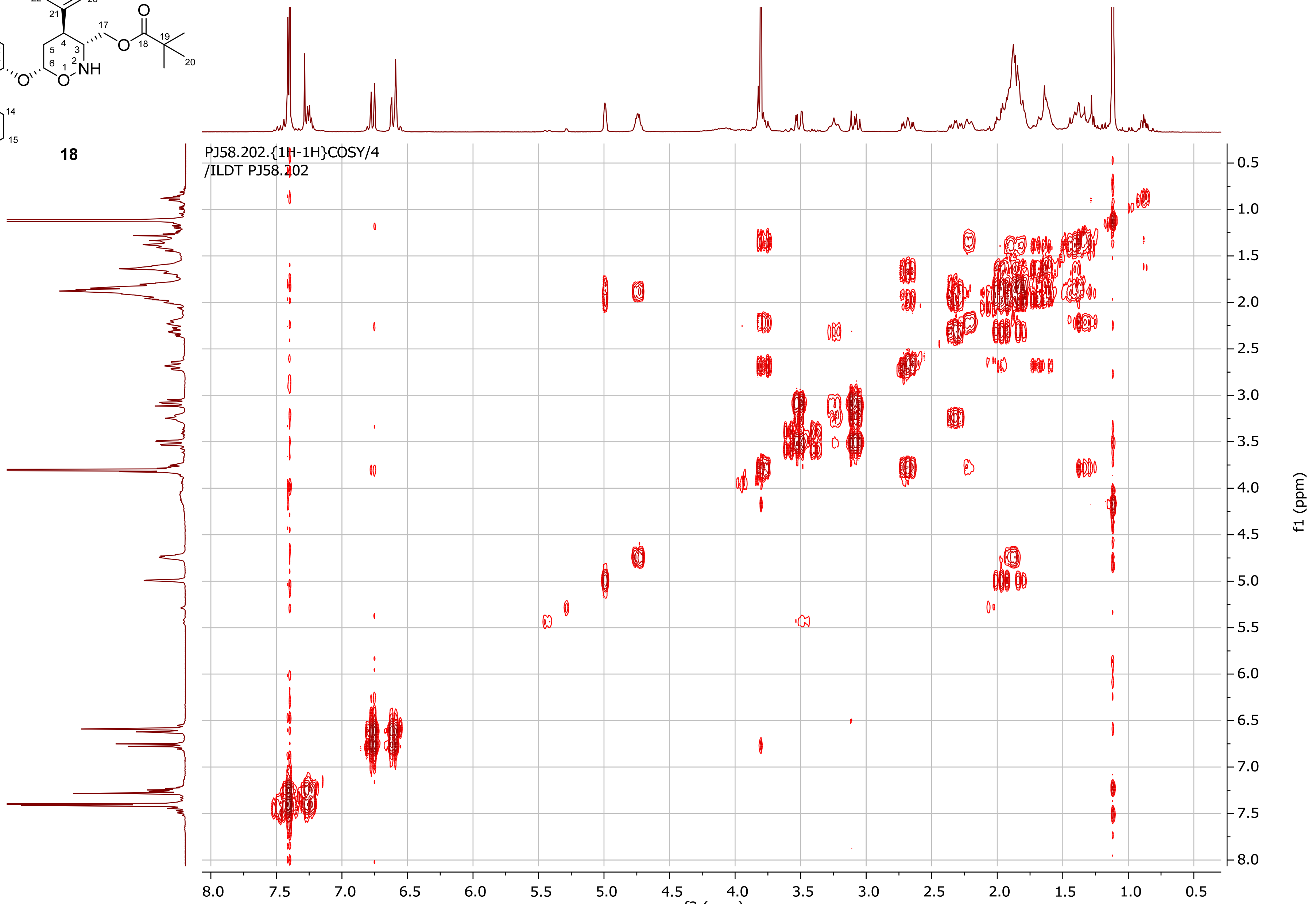

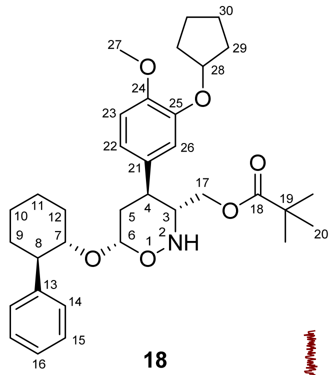

18

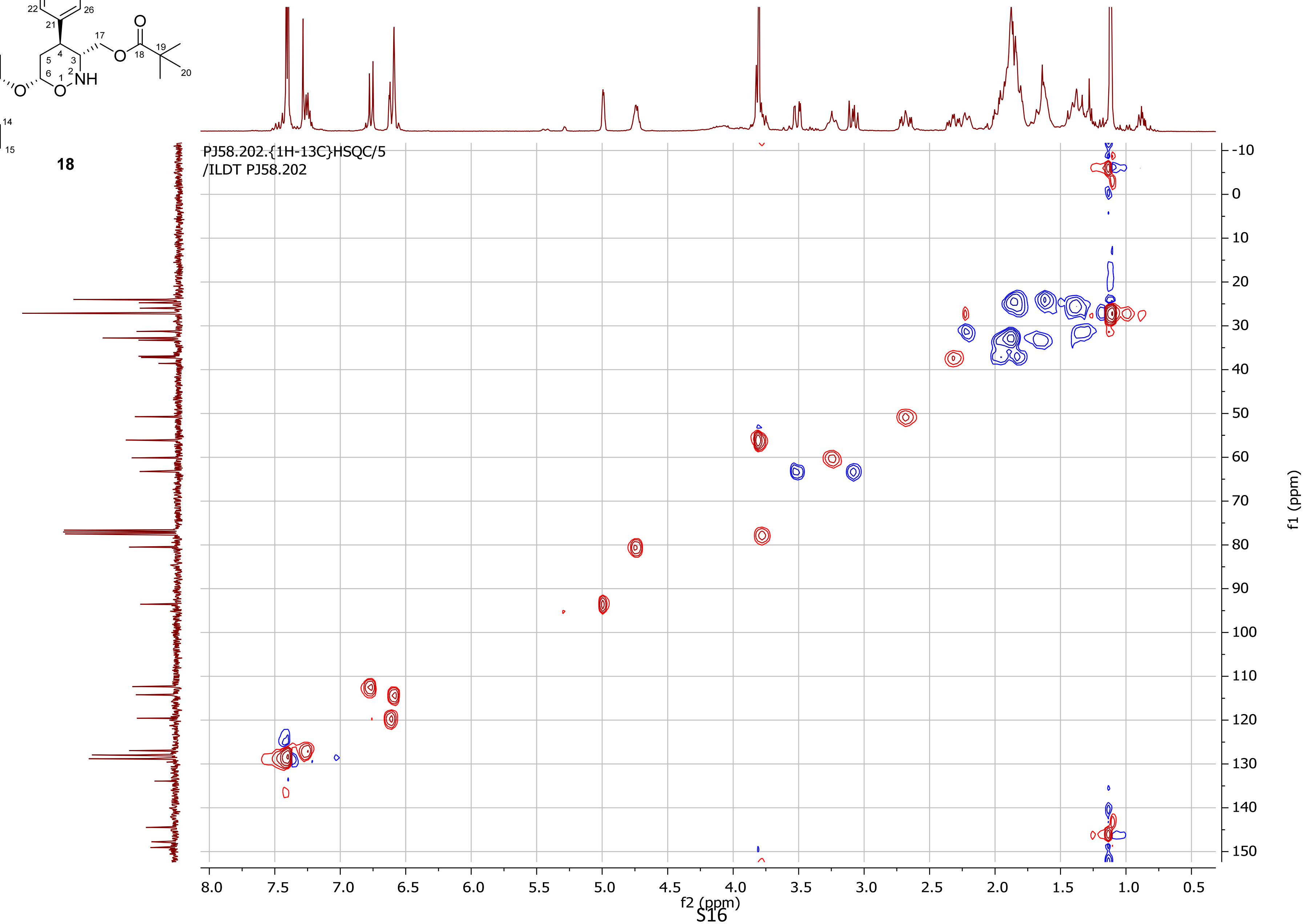

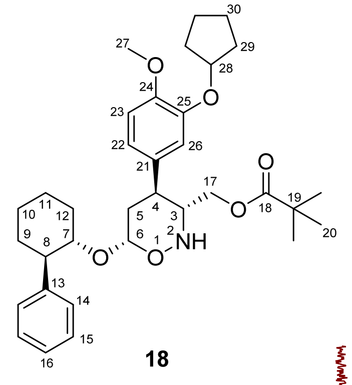

18

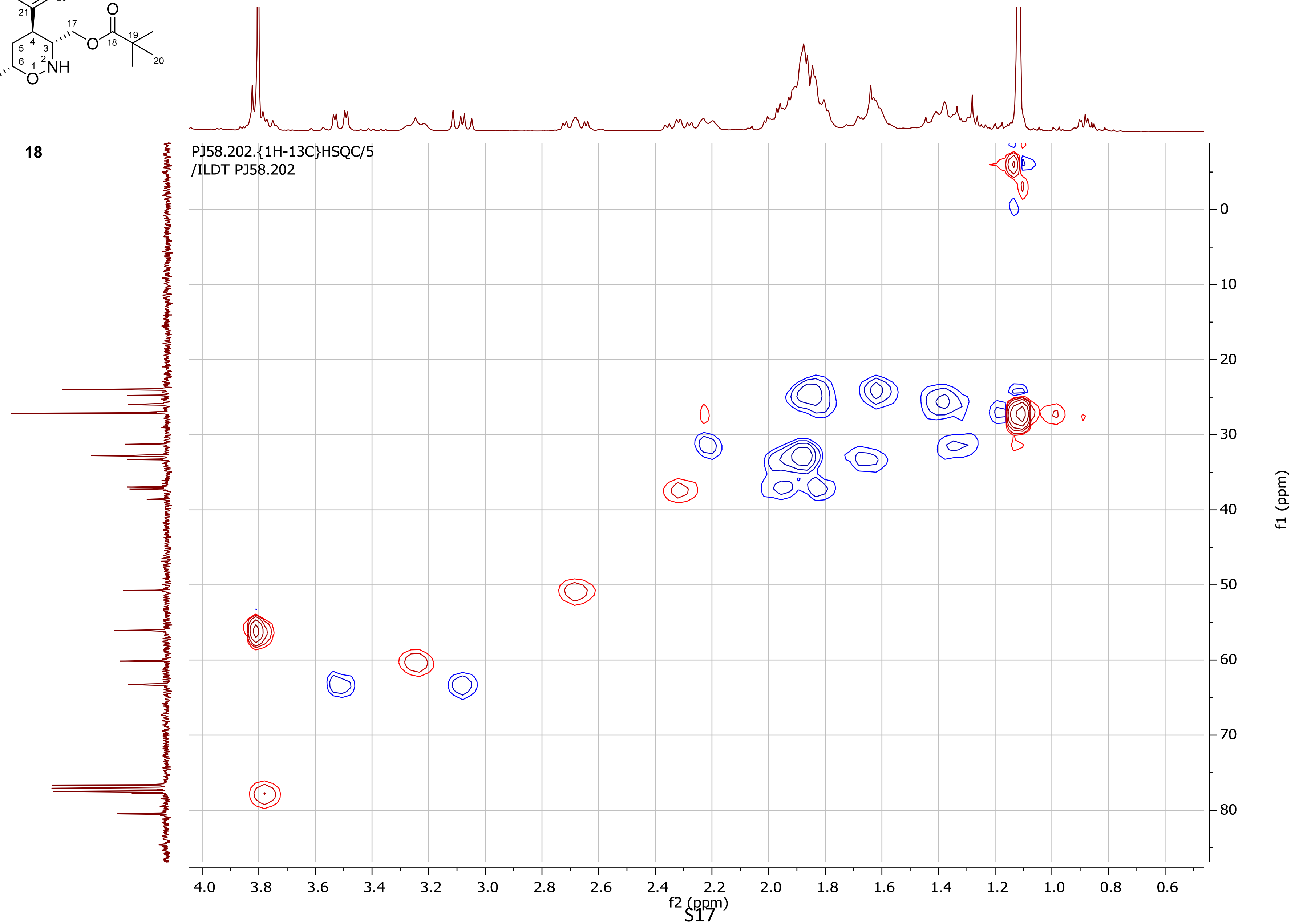

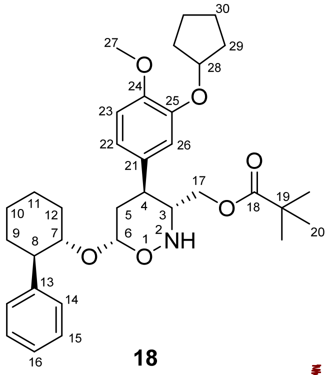
27
30
29
28
24
25
23
22
26
21
17
19
18
20
4
5
3
11
10
12
2
9
8
7
6
1
NH
13
14
15
16

18

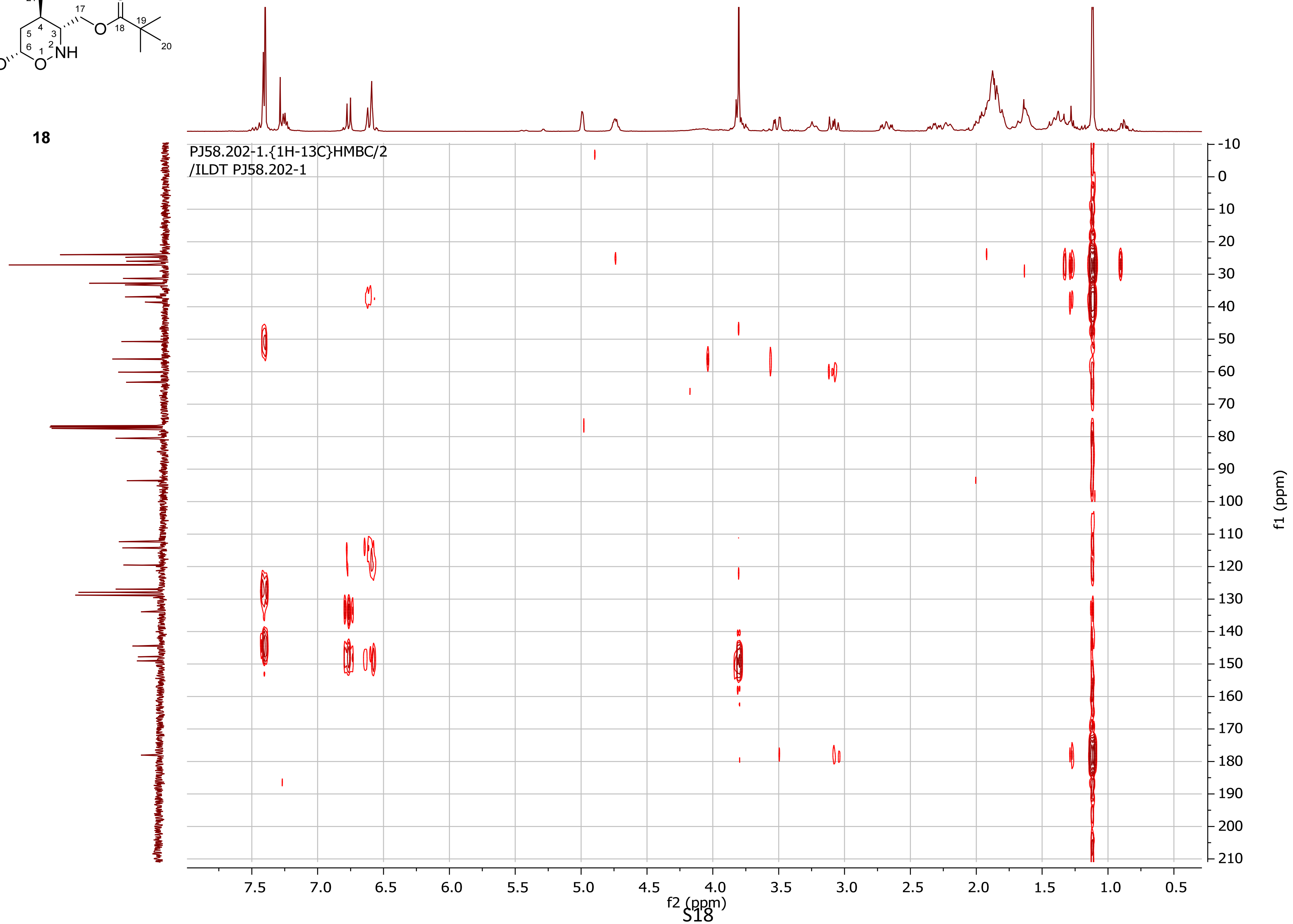
PJ58.202-1.{1H-13C}HMBC/2
/ILDT PJ58.202-1
f2 (ppm)
f1 (ppm)

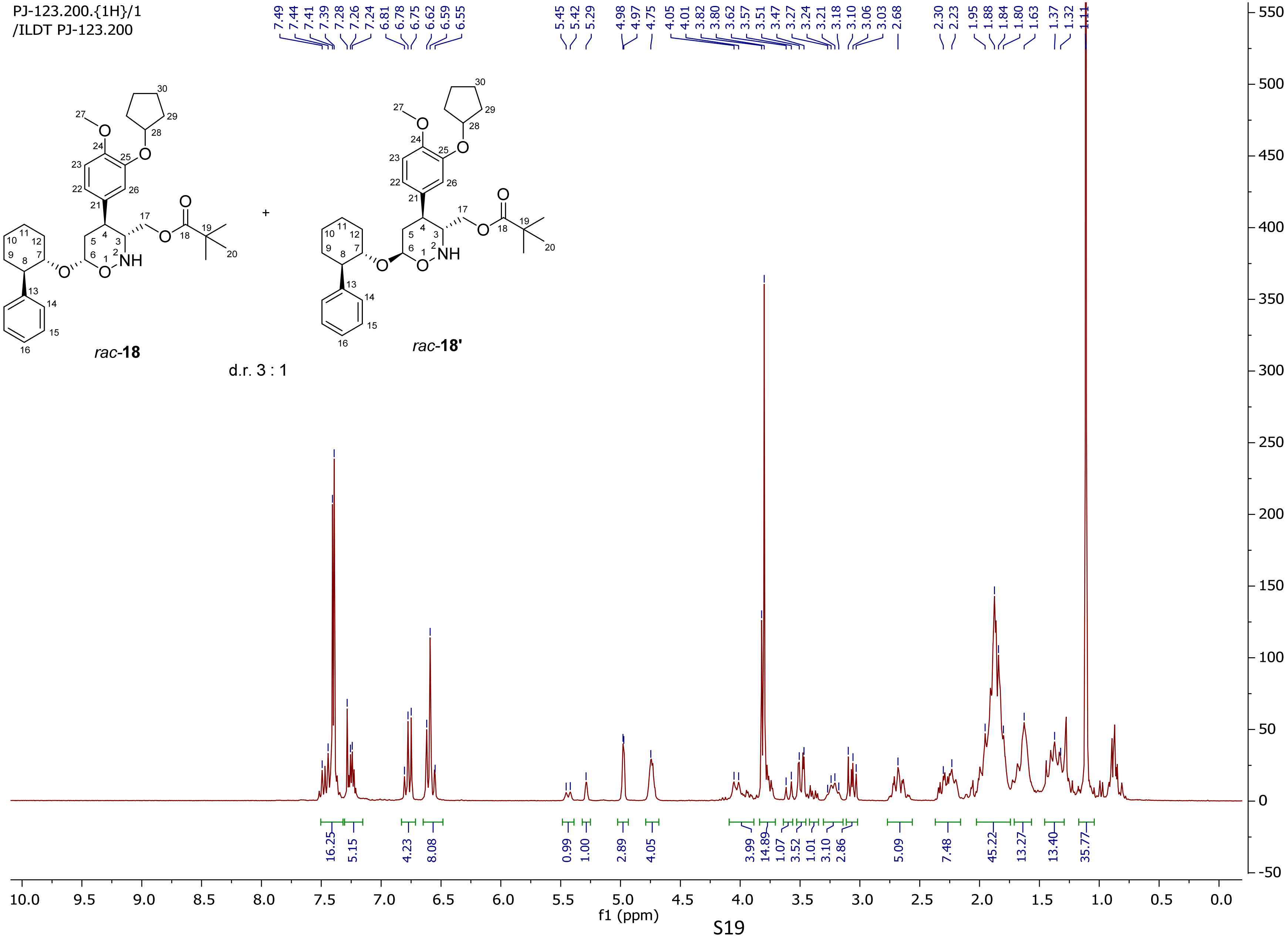
PJ-123.200.{1H}/1
/ILDT PJ-123.200
rac-18
rac-18'
d.r. 3 : 1
f1 (ppm)

PJ-116.2.{1H}/1
/TERN i6514

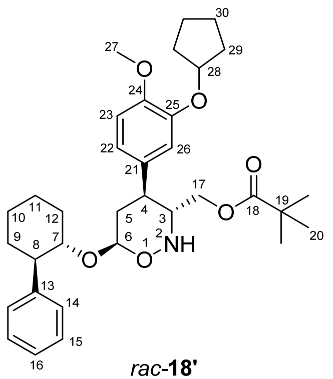

rac-**18'**

+

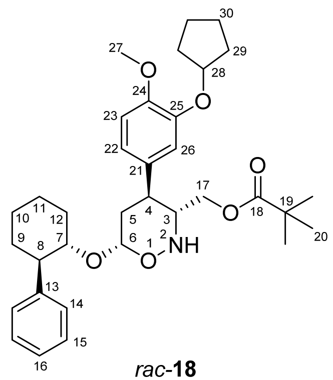

rac-**18**

d.r. 7 : 1

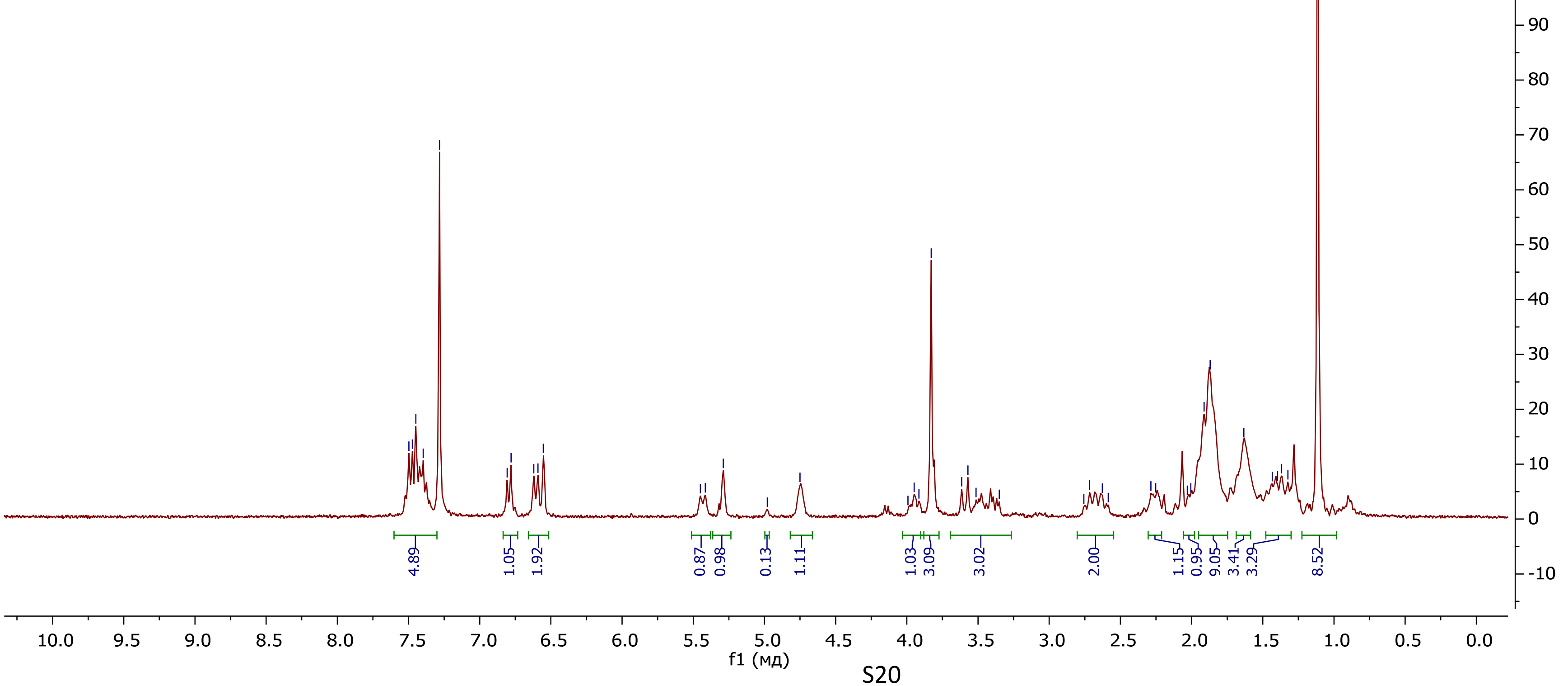

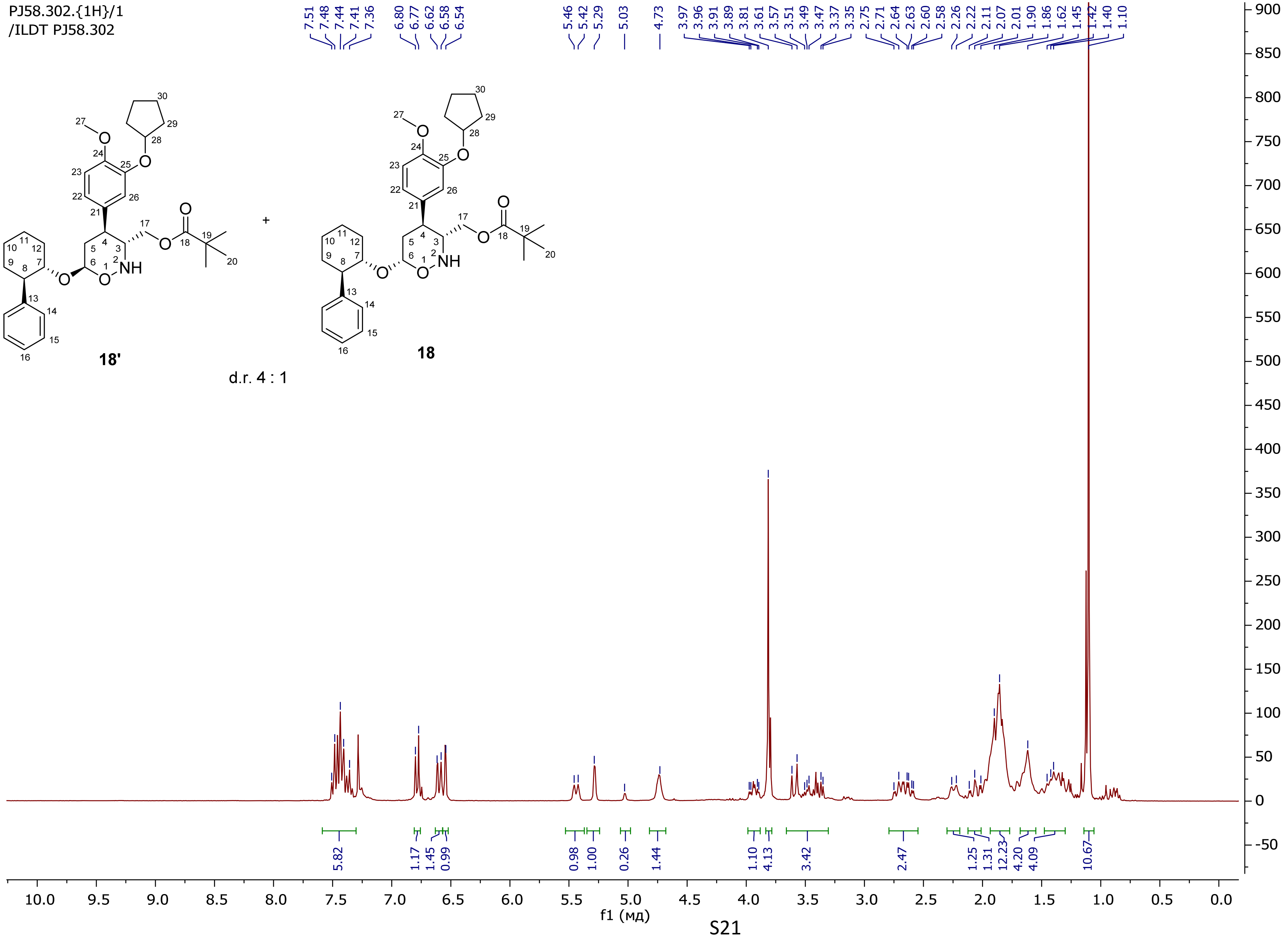
PJ58.302.{1H}/1
/ILDT PJ58.302
7.51 7.48 7.44 7.41 7.36 6.80 6.77 6.62 6.58 6.54 5.46 5.42 5.29 5.03 4.73 3.97 3.96 3.91 3.89 3.81 3.61 3.57 3.51 3.49 3.47 3.37 3.35 2.75 2.71 2.64 2.63 2.60 2.58 2.26 2.22 2.11 2.07 2.01 1.90 1.86 1.62 1.45 1.42 1.40 1.10
18'
+
18
d.r. 4 : 1
5.82 1.17 1.45 0.99 0.98 1.00 0.26 1.44 1.10 4.13 3.42 2.47 1.25 1.31 12.23 4.20 4.09 10.67
f1 (мд)

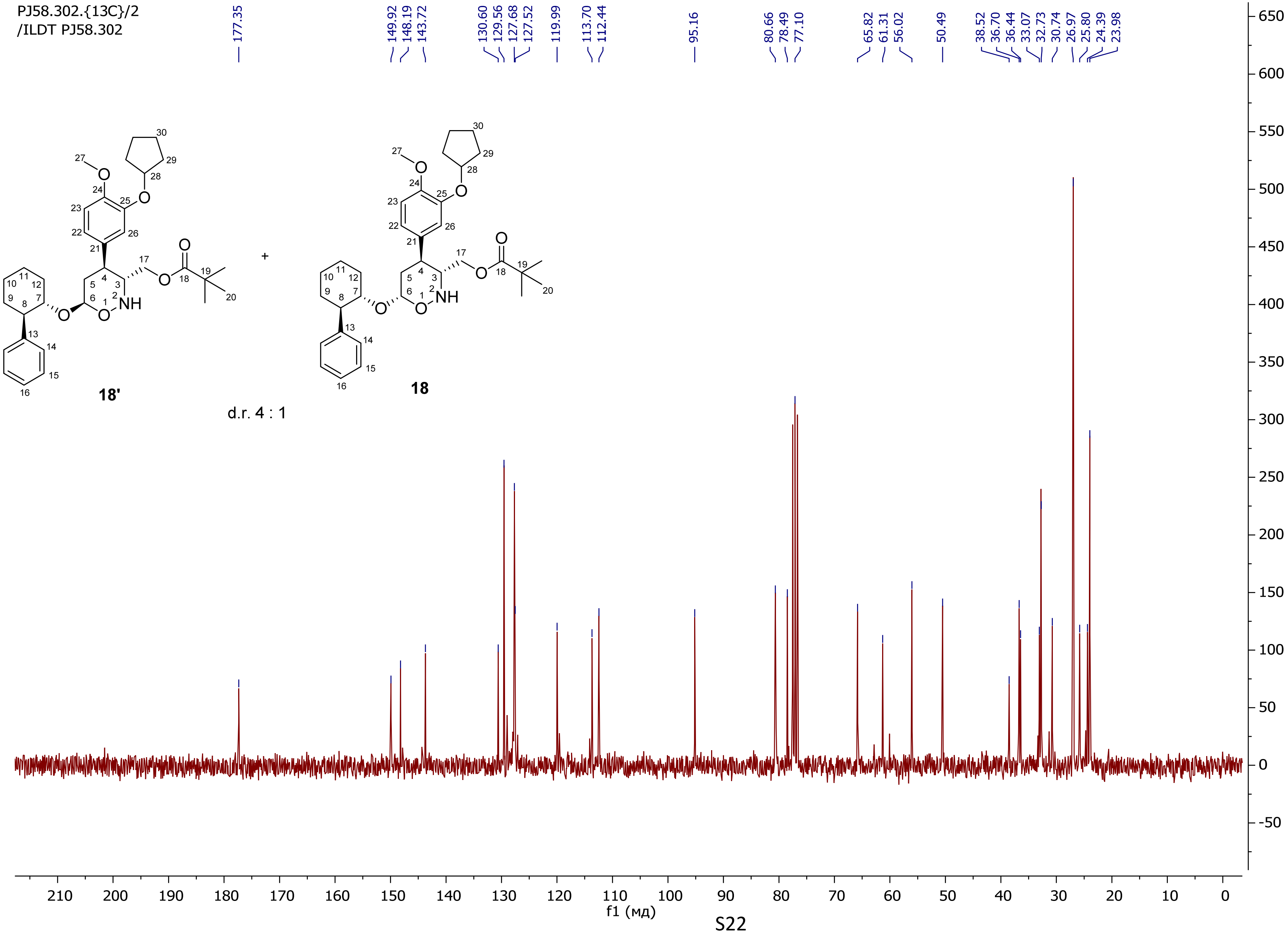
PJ58.302.{13C}/2
/ILDT PJ58.302
177.35
149.92
148.19
143.72
130.60
129.56
127.68
127.52
119.99
113.70
112.44
95.16
80.66
78.49
77.10
65.82
61.31
56.02
50.49
38.52
36.70
36.44
33.07
32.73
30.74
26.97
25.80
24.39
23.98
18'
+
18
d.r. 4 : 1
f1 (мд)

PJ58.302.{13C}deptsp135/3
/ILDT PJ58.302

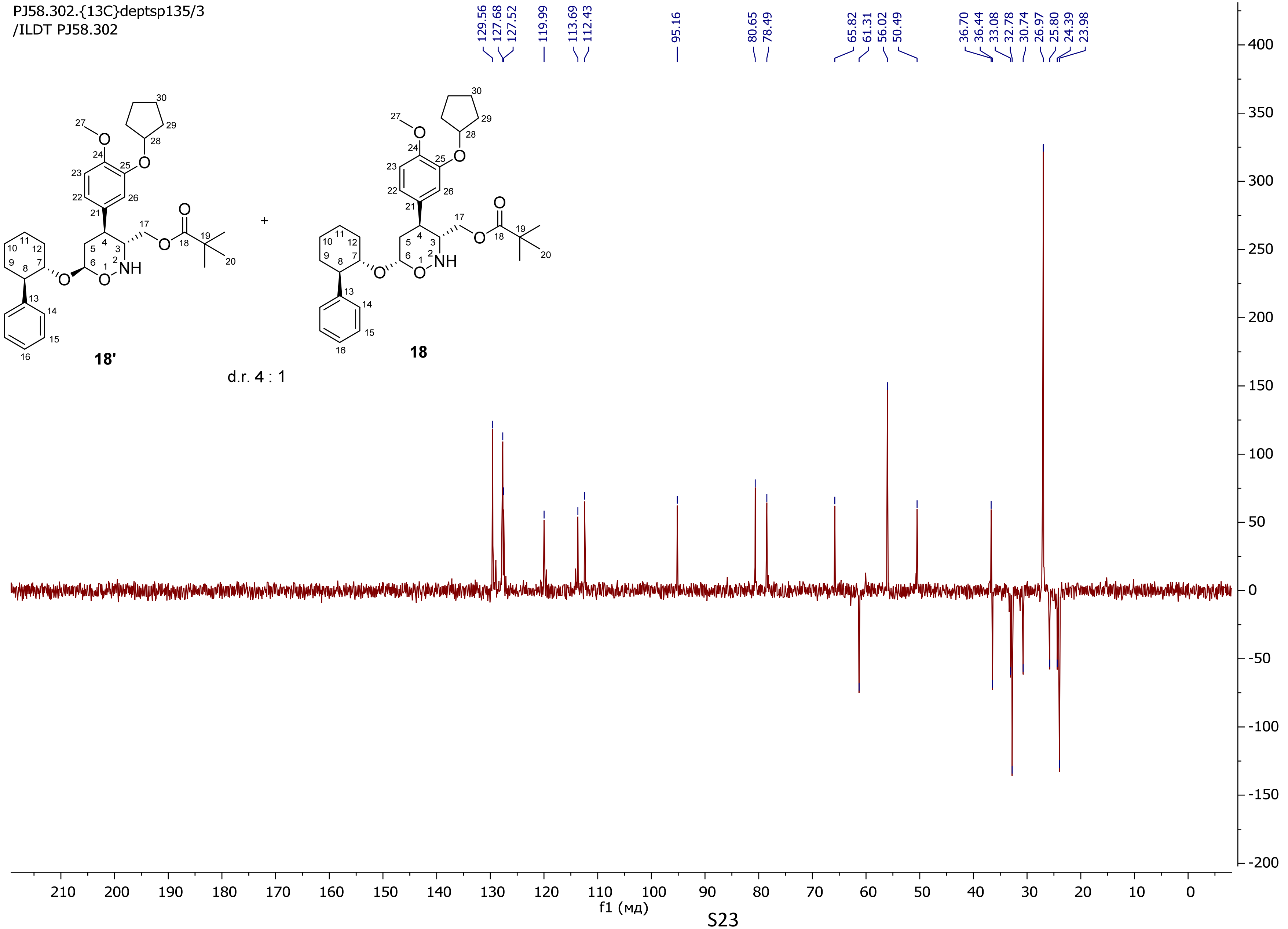

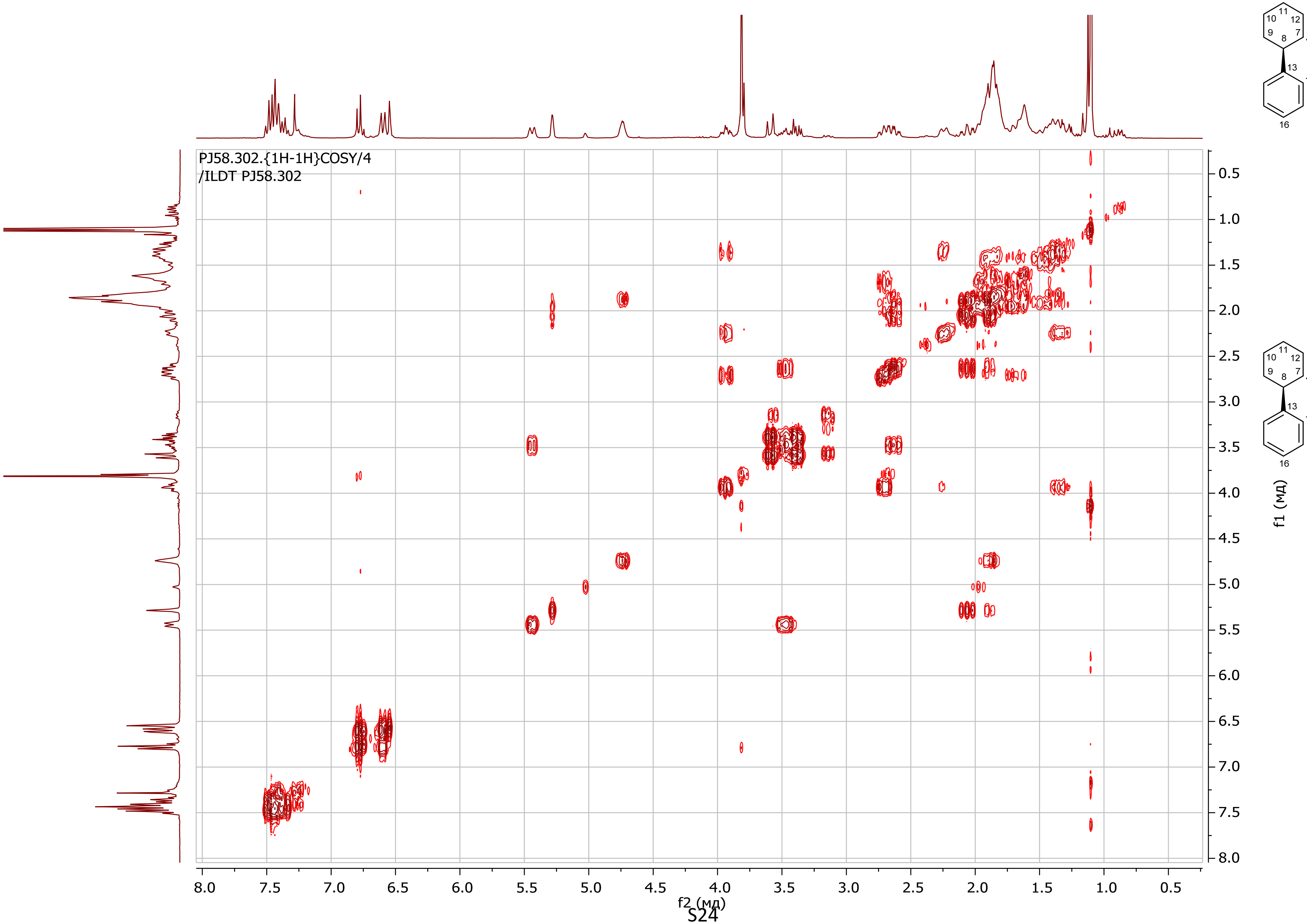
PJ58.302.{1H-1H}COSY/4
/ILDT PJ58.302
f2 (мд)
f1 (мд)
8.0 7.5 7.0 6.5 6.0 5.5 5.0 4.5 4.0 3.5 3.0 2.5 2.0 1.5 1.0 0.5

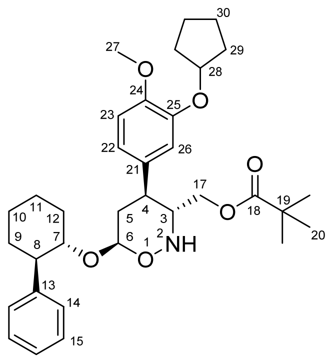
27
24
25
23
22
26
21
28
29
30
17
18
19
20
4
3
5
6
2
1
NH
10
11
12
9
8
7
13
14
15
16

18'

+

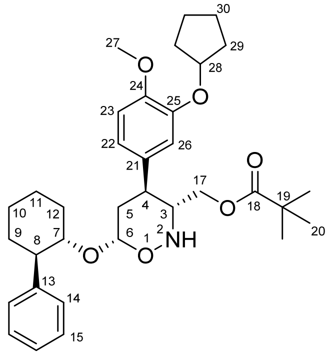
27
24
25
23
22
26
21
28
29
30
17
18
19
20
4
3
5
6
2
1
NH
10
11
12
9
8
7
13
14
15
16

18

d.r. 4 : 1

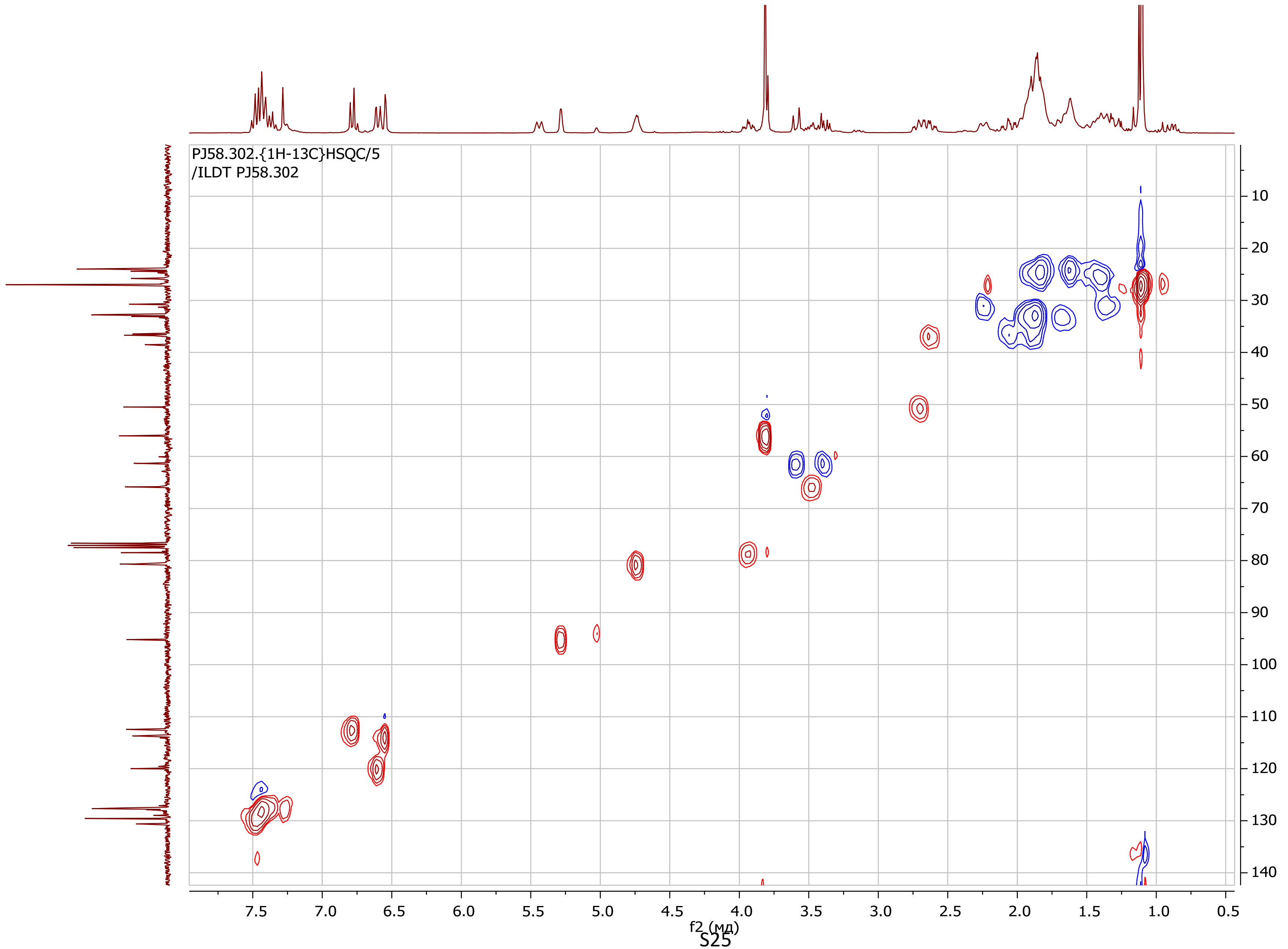

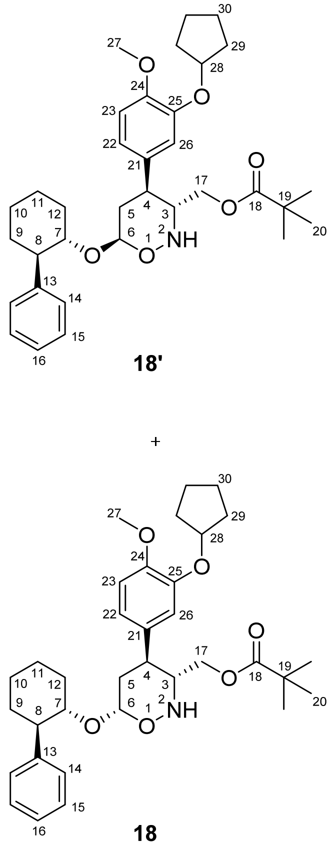

18' + 18

d.r. 4 : 1

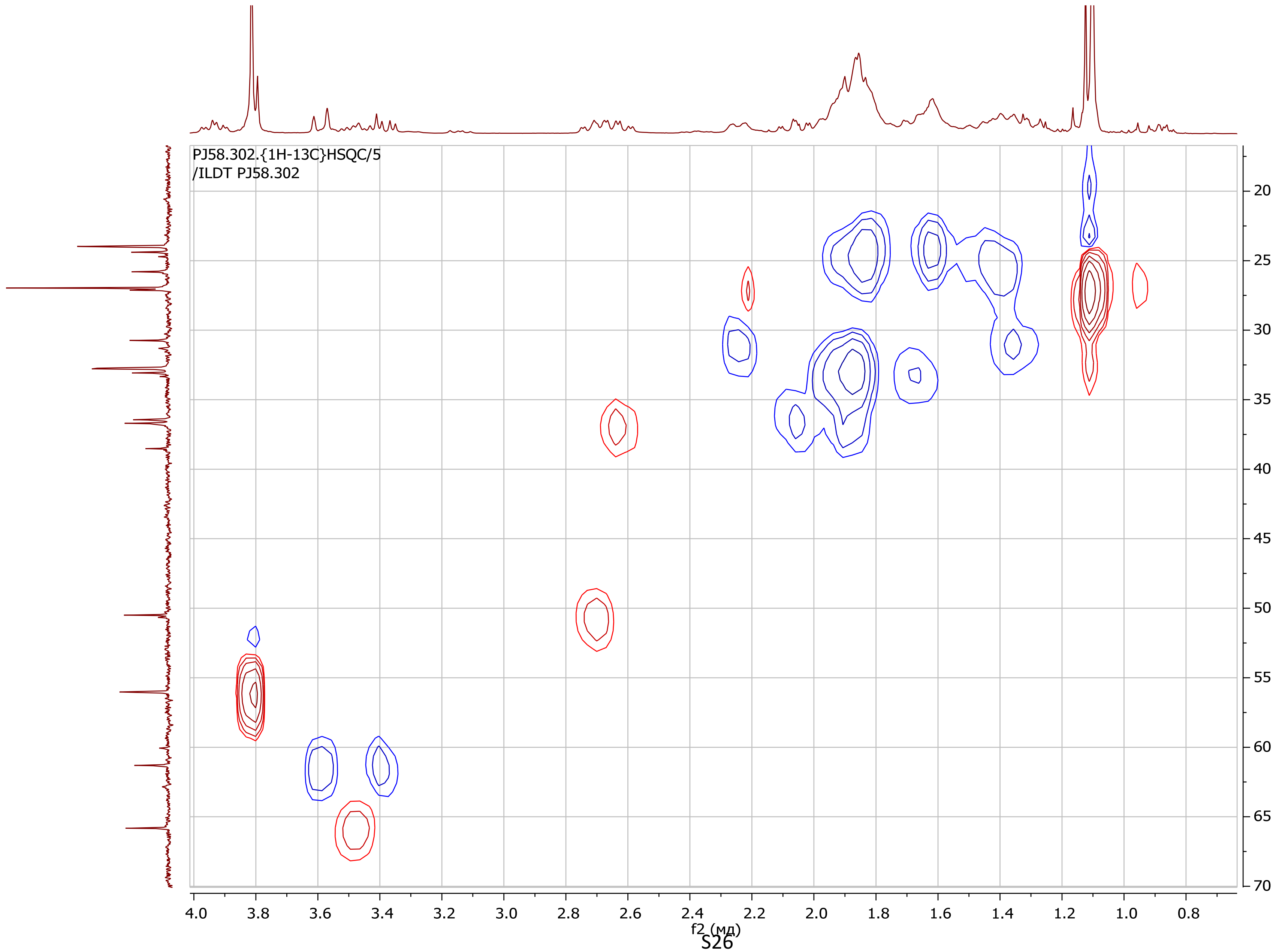

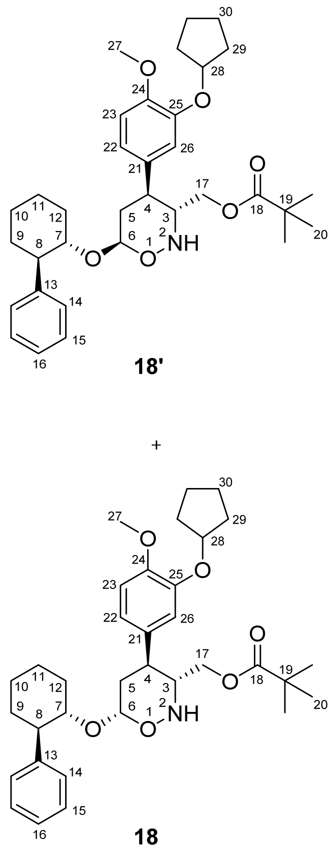

18' + 18

d.r. 4 : 1

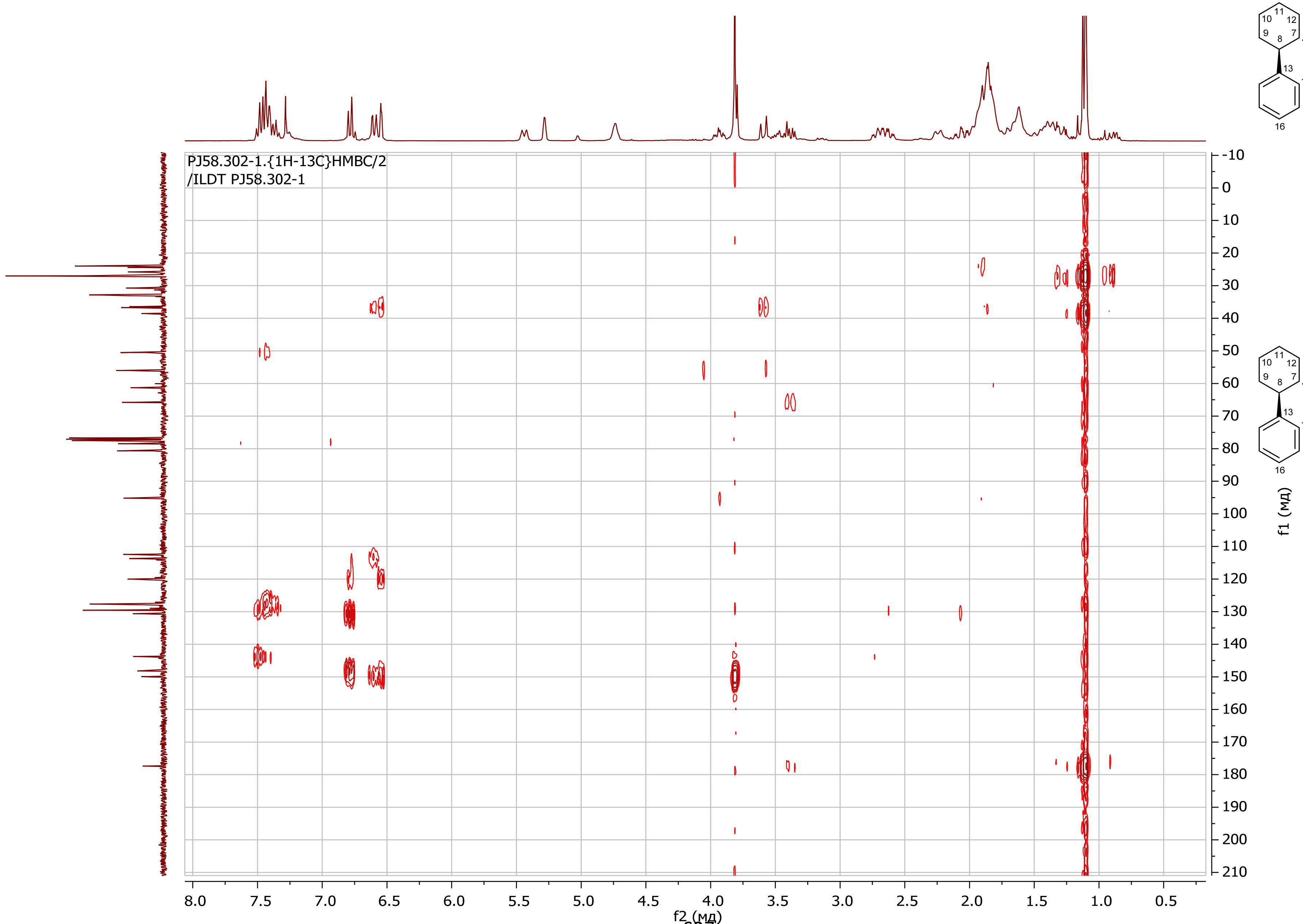

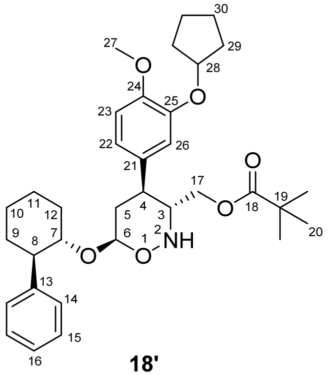

18'

+

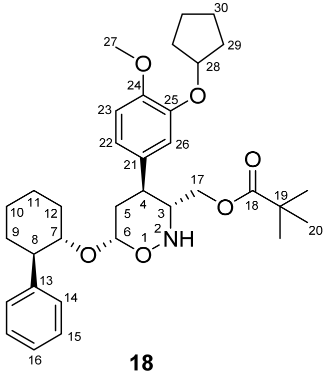

18

d.r. 4 : 1

PJ-59.204.{1H}/1
/ILDT PJ-59.204

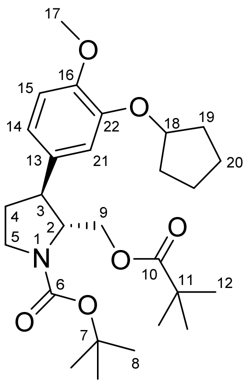

(-)-**19**
r.t.

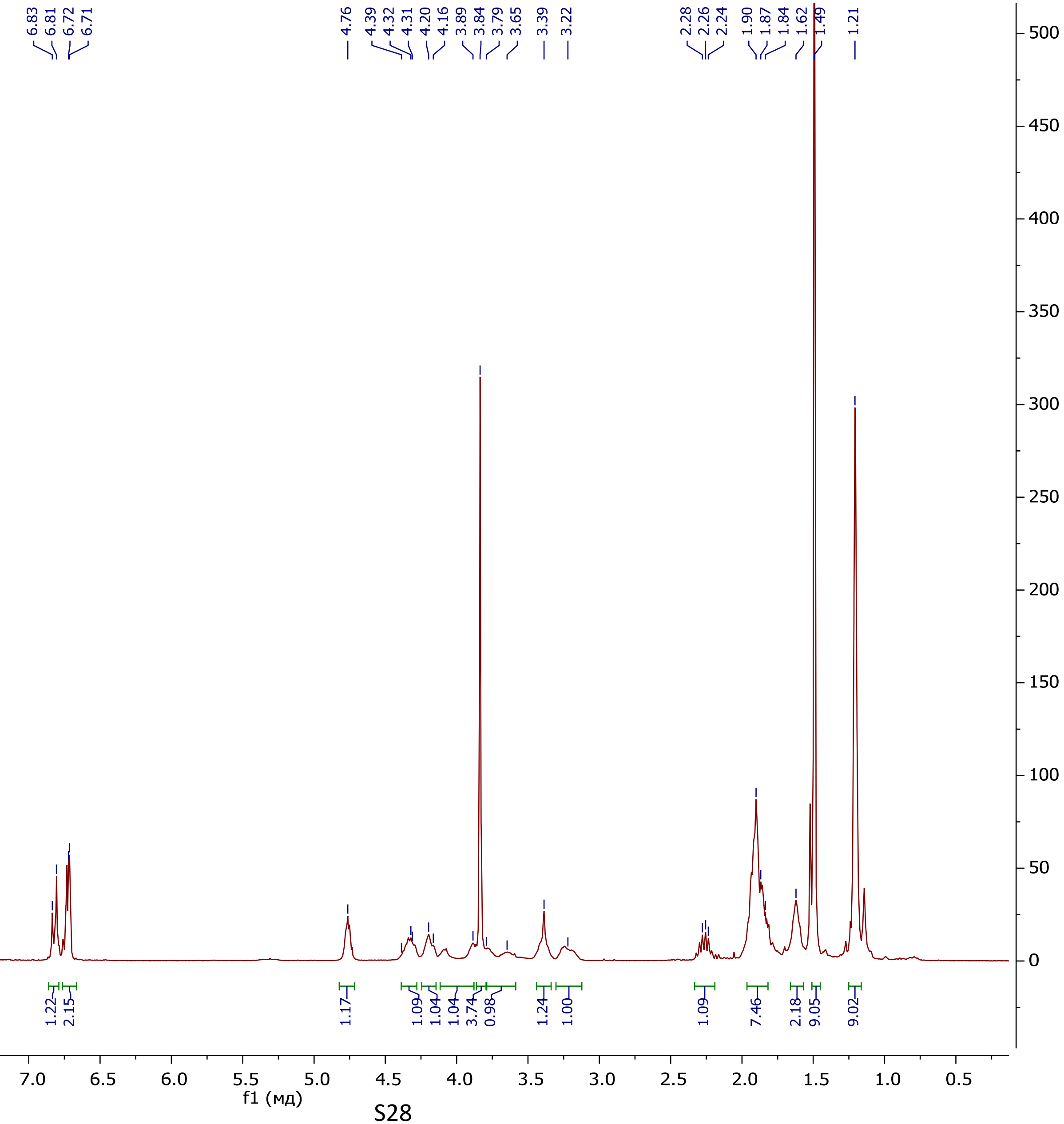

PJ-59.204-320K.{1H}/6
/ILDT PJ-59.204-320K

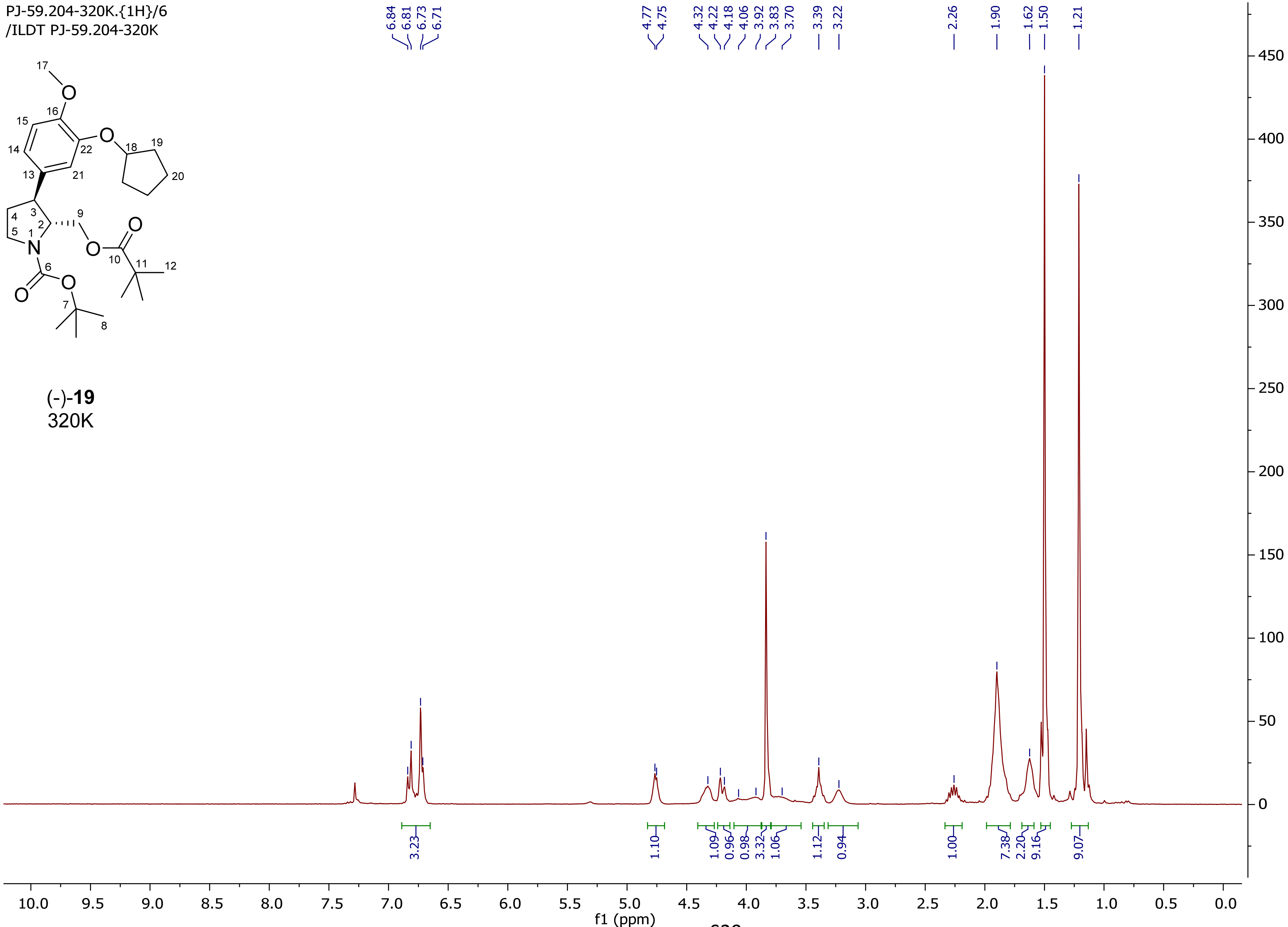

(-)-**19**
320K

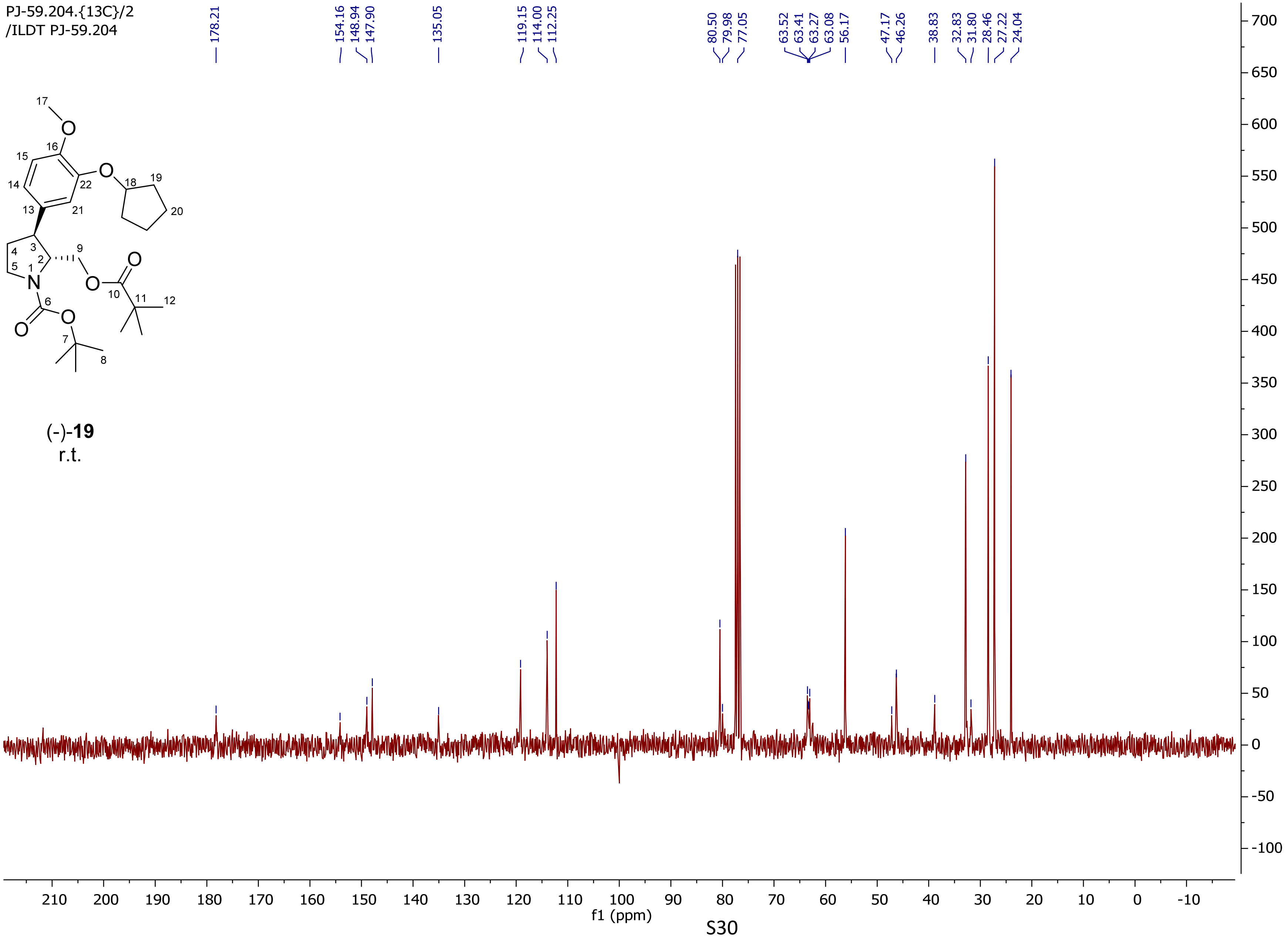
PJ-59.204.{13C}/2
/ILDT PJ-59.204
178.21
154.16
148.94
147.90
135.05
119.15
114.00
112.25
80.50
79.98
77.05
63.52
63.41
63.27
63.08
56.17
47.17
46.26
38.83
32.83
31.80
28.46
27.22
24.04
(-)-19
r.t.
f1 (ppm)

PJ-59.204.{13C}deptsp135/3
/ILDT PJ-59.204

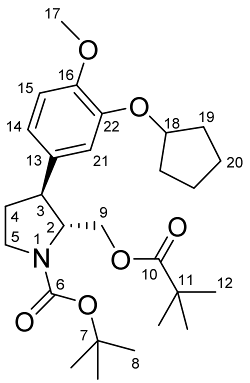

(-)-**19**
r.t.

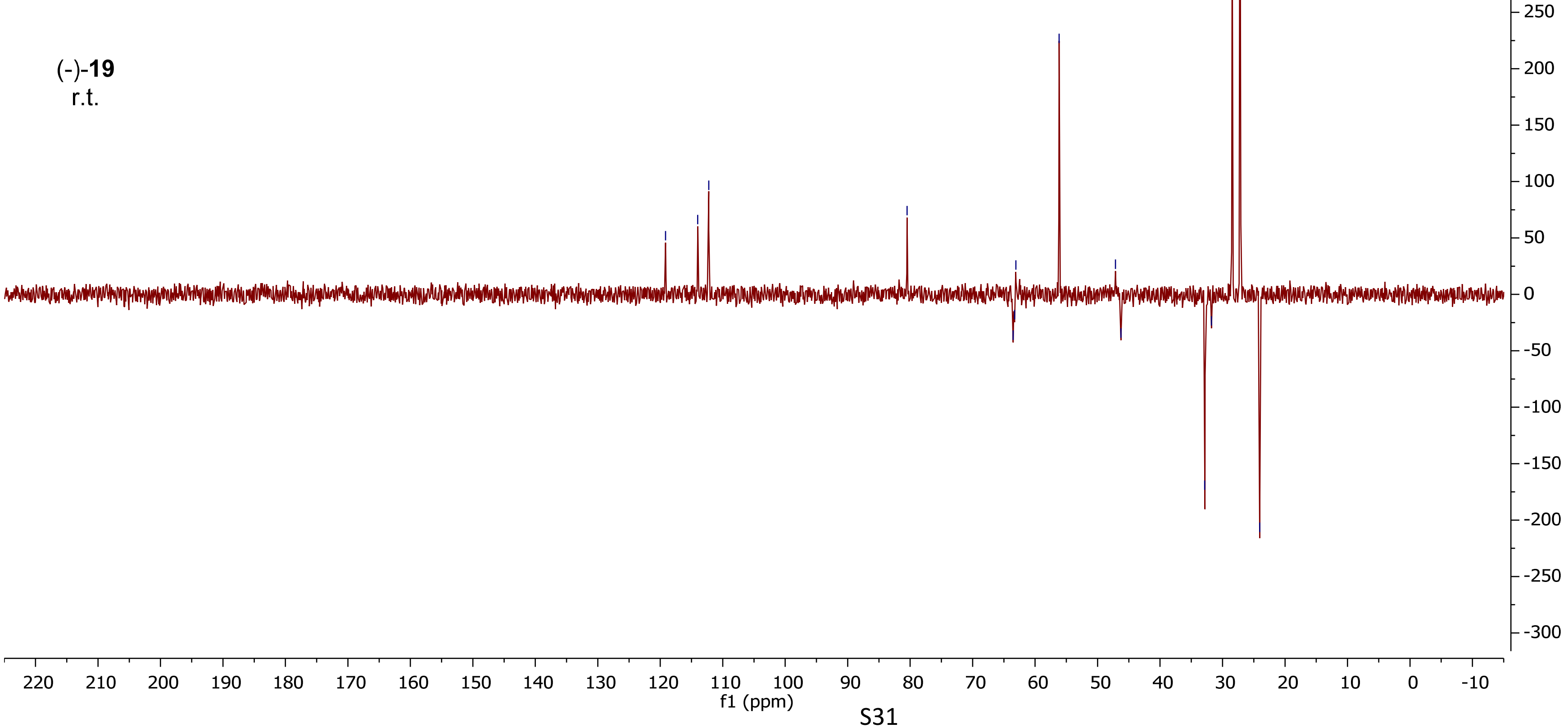

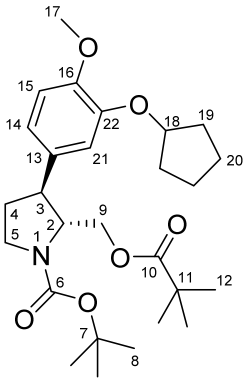
17
16
15
14
22
18
19
20
13
21
3
4
5
2
1
9
10
11
12
6
7
8

(-)-**19**
r.t.

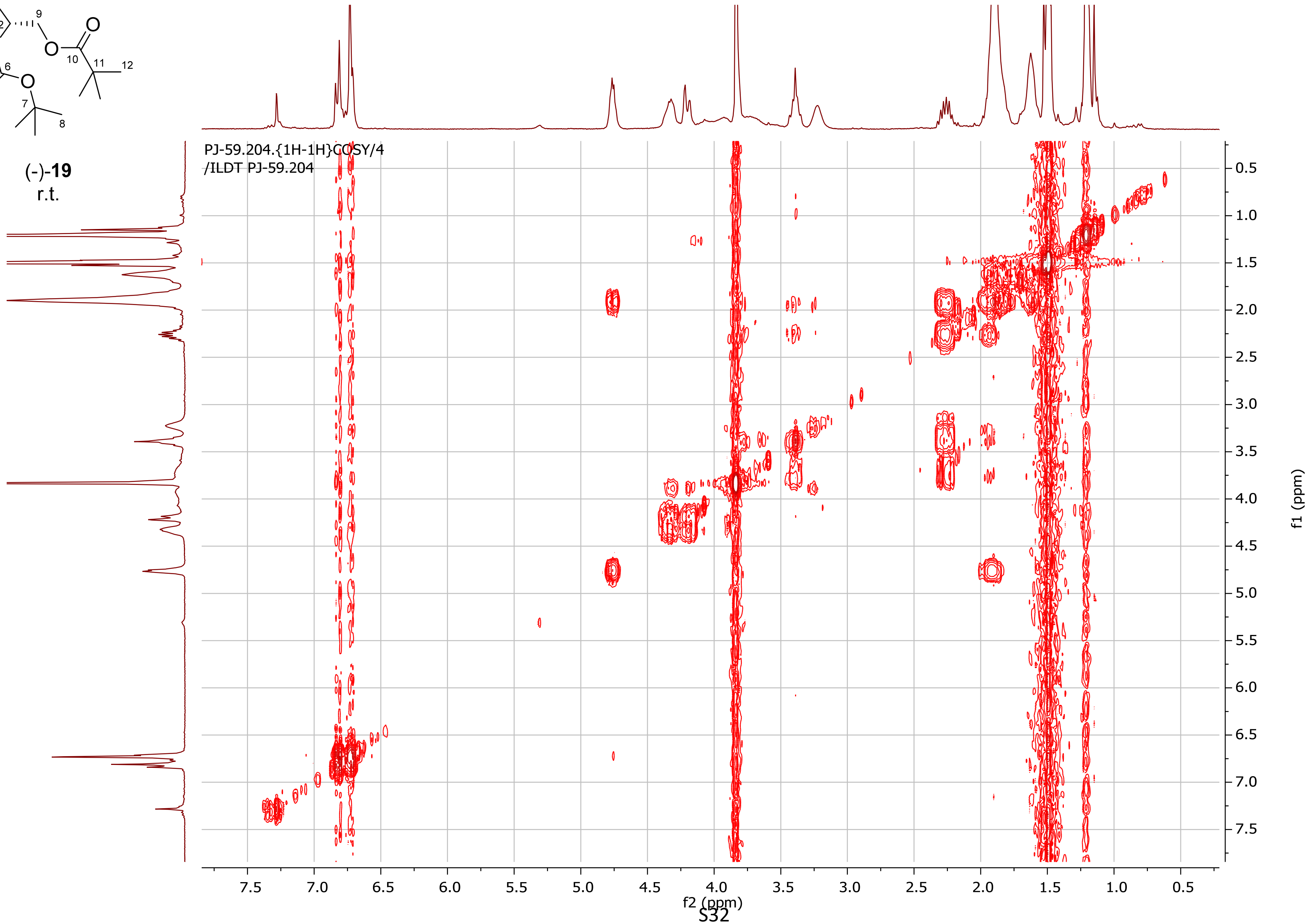
PJ-59.204.{1H-1H}COSY/4
/ILDT PJ-59.204
f2 (ppm)
f1 (ppm)

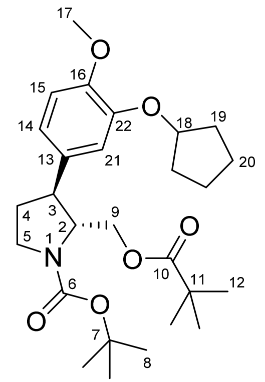

17
16
15
14
13
22
21
18
19
20
3
4
5
2
1
9
N
6
7
8
10
11
12

(-)-**19**
r.t.

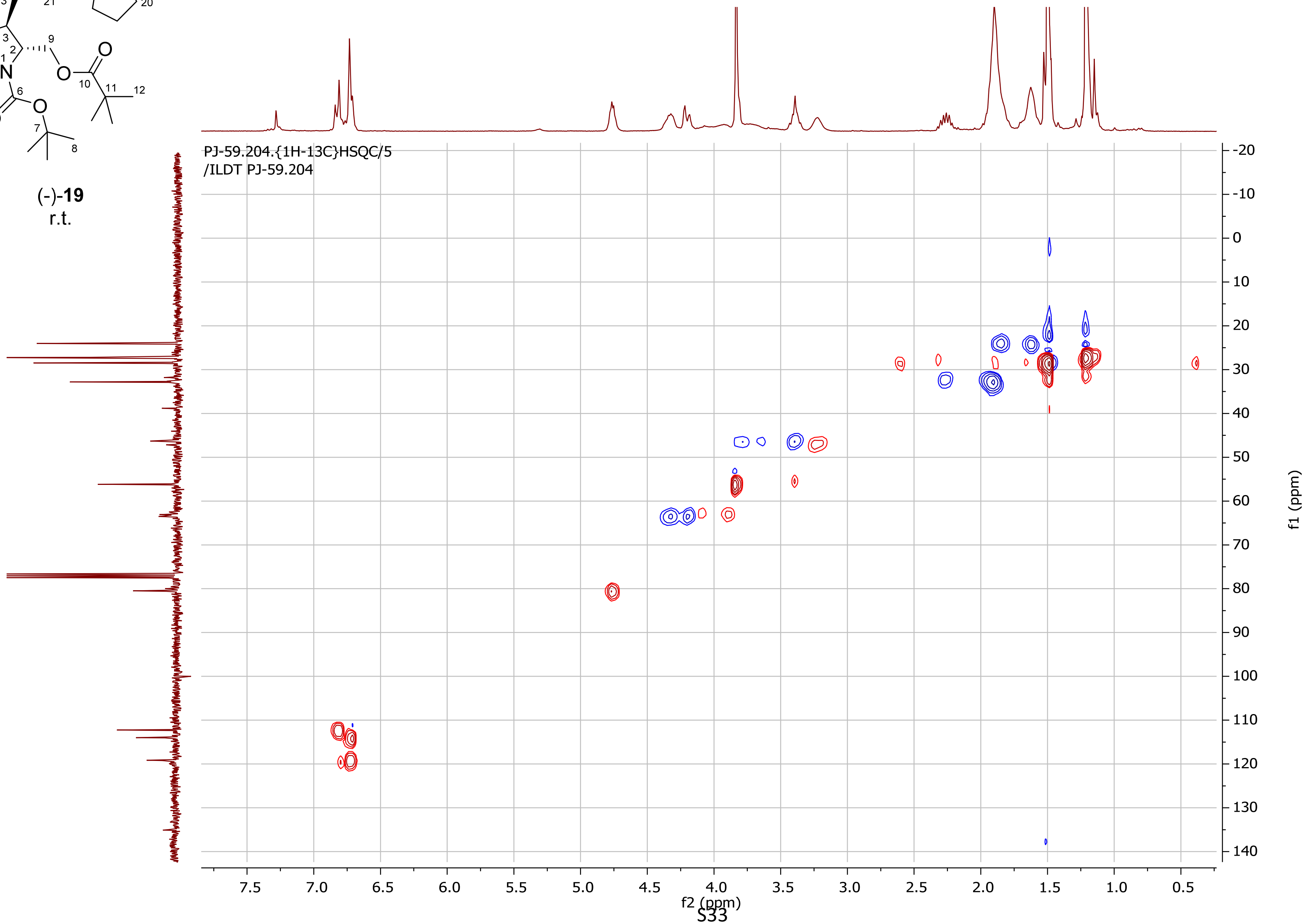

PJ-59.204.{1H-13C}HSQC/5
/ILDT PJ-59.204
f2 (ppm)
f1 (ppm)

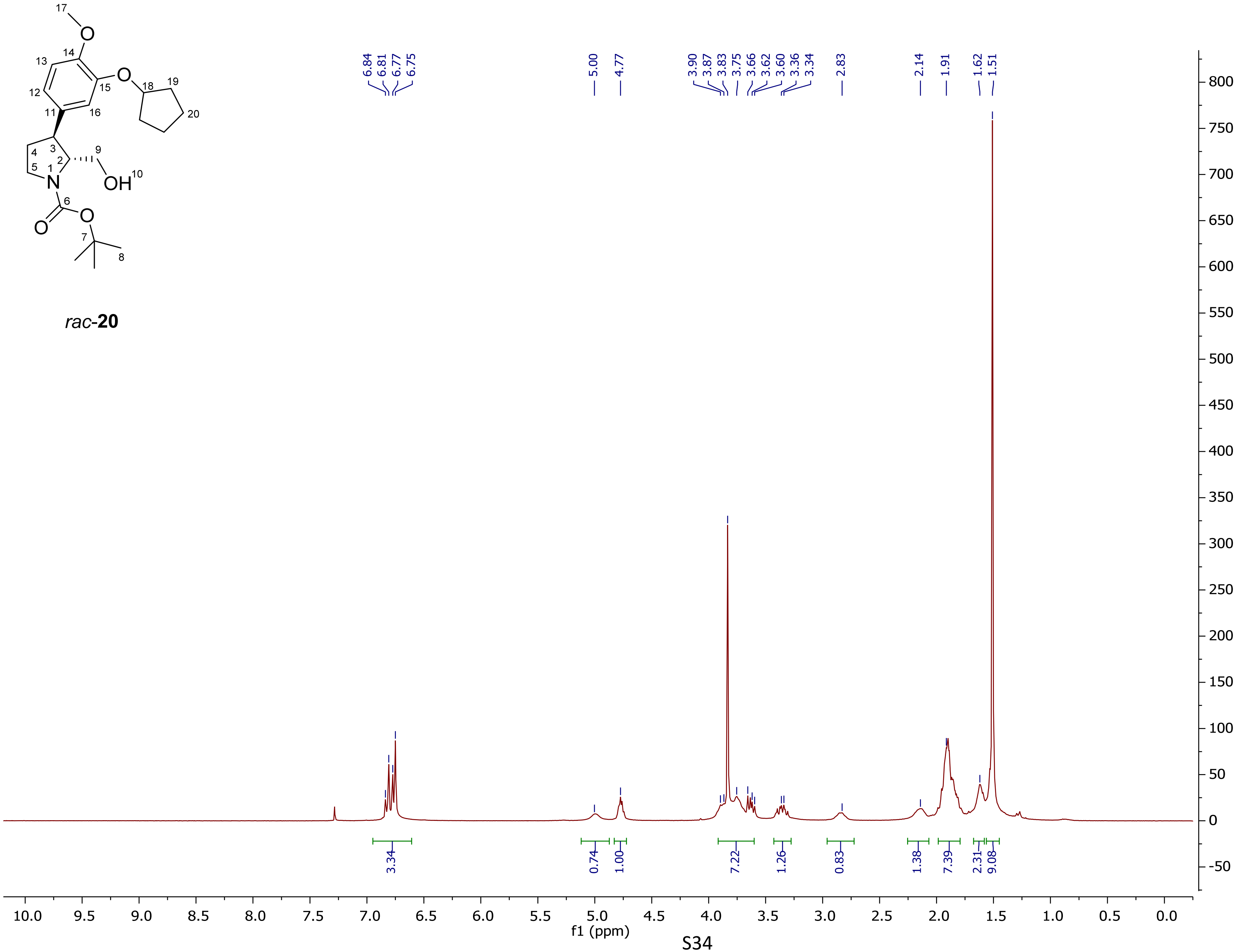

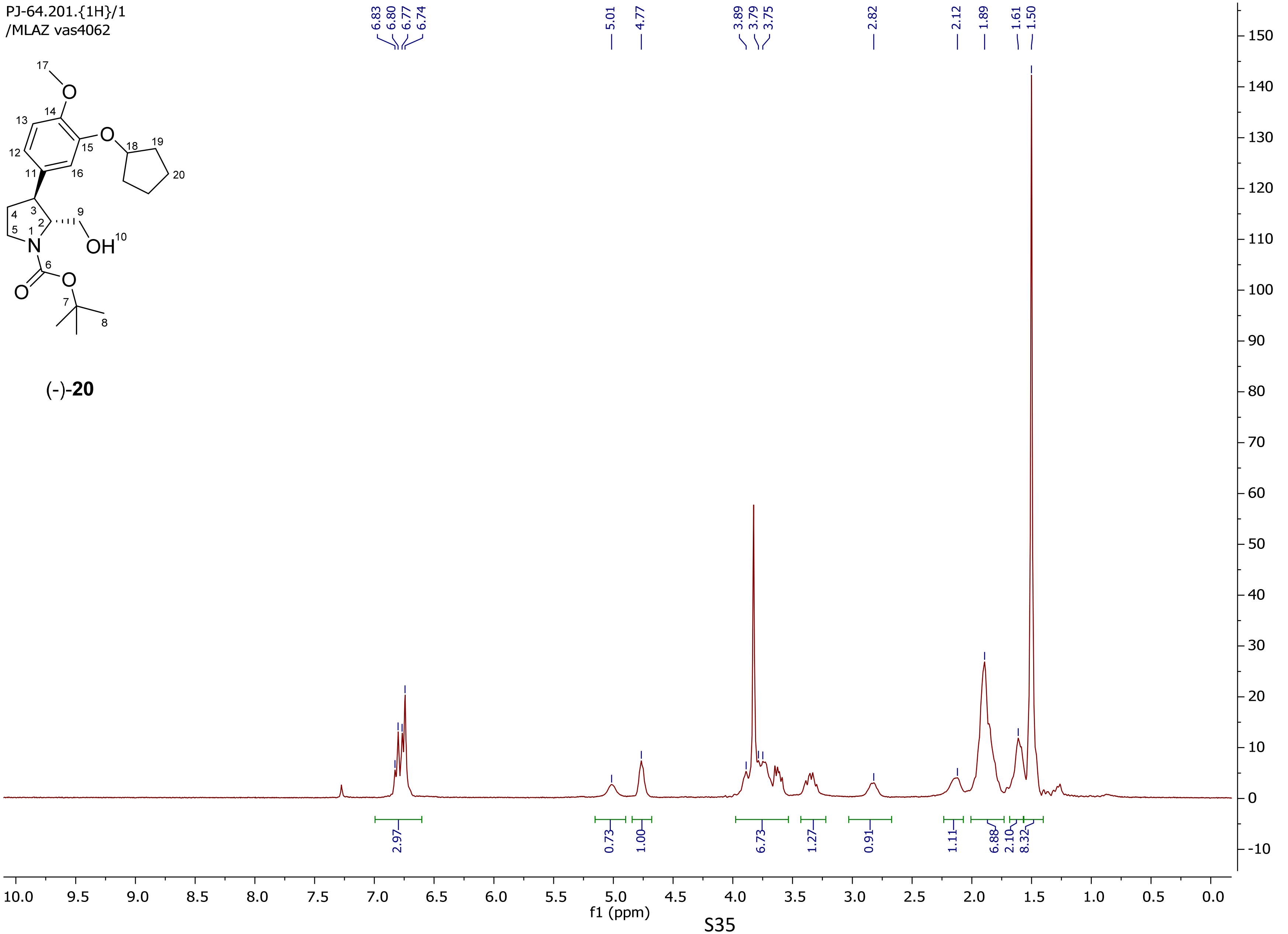
PJ-64.201.{1H}/1
/MLAZ vas4062
(-)-20
6.83
6.80
6.77
6.74
5.01
4.77
3.89
3.79
3.75
2.82
2.12
1.89
1.61
1.50
2.97
0.73
1.00
6.73
1.27
0.91
1.11
6.88
2.10
8.32
f1 (ppm)

PJ64.201-2.{13C}/2
/ILDT PJ64.201-2

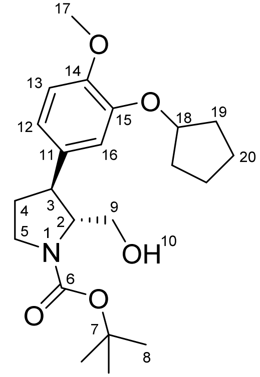

(-)-**20**

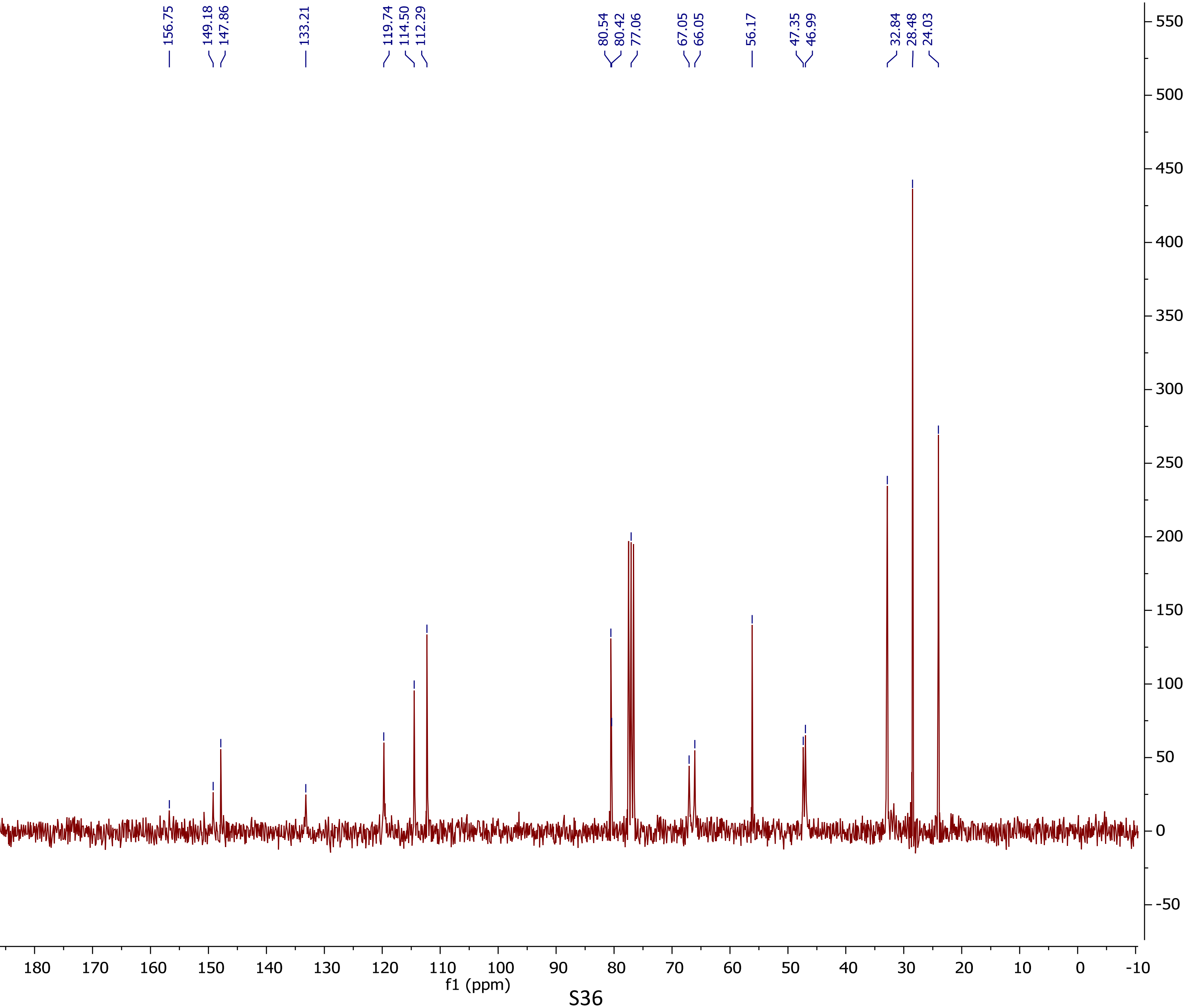

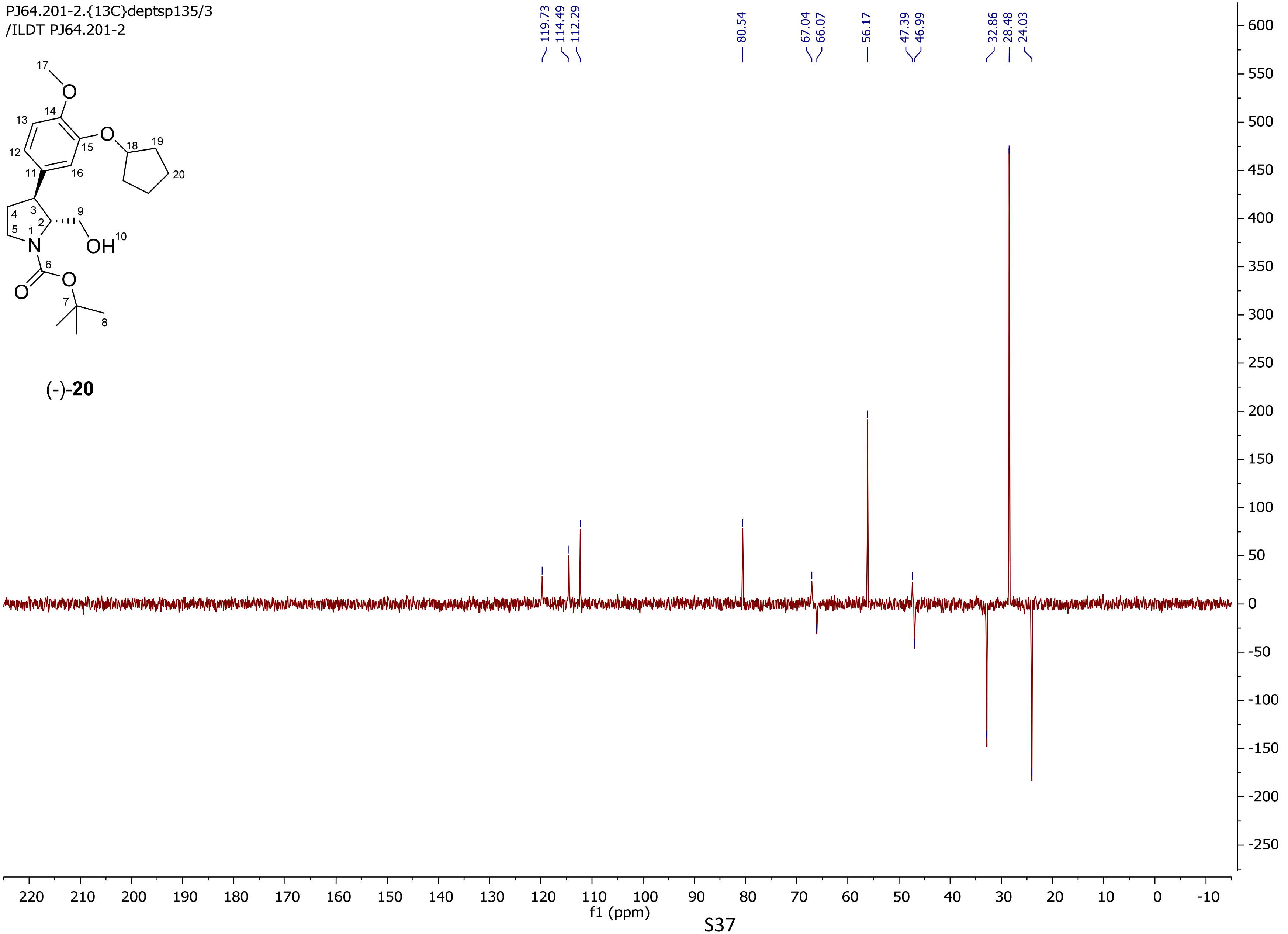
PJ64.201-2.{13C}deptsp135/3
/ILDT PJ64.201-2
(-)-20
119.73
114.49
112.29
80.54
67.04
66.07
56.17
47.39
46.99
32.86
28.48
24.03
f1 (ppm)

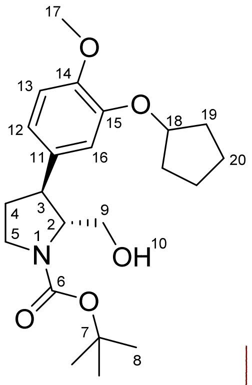
17
O
14
13
O
15
18
19
12
20
11
16
3
9
4
2
5
1
N
OH
10
6
O
7
O
8

(-)-**20**

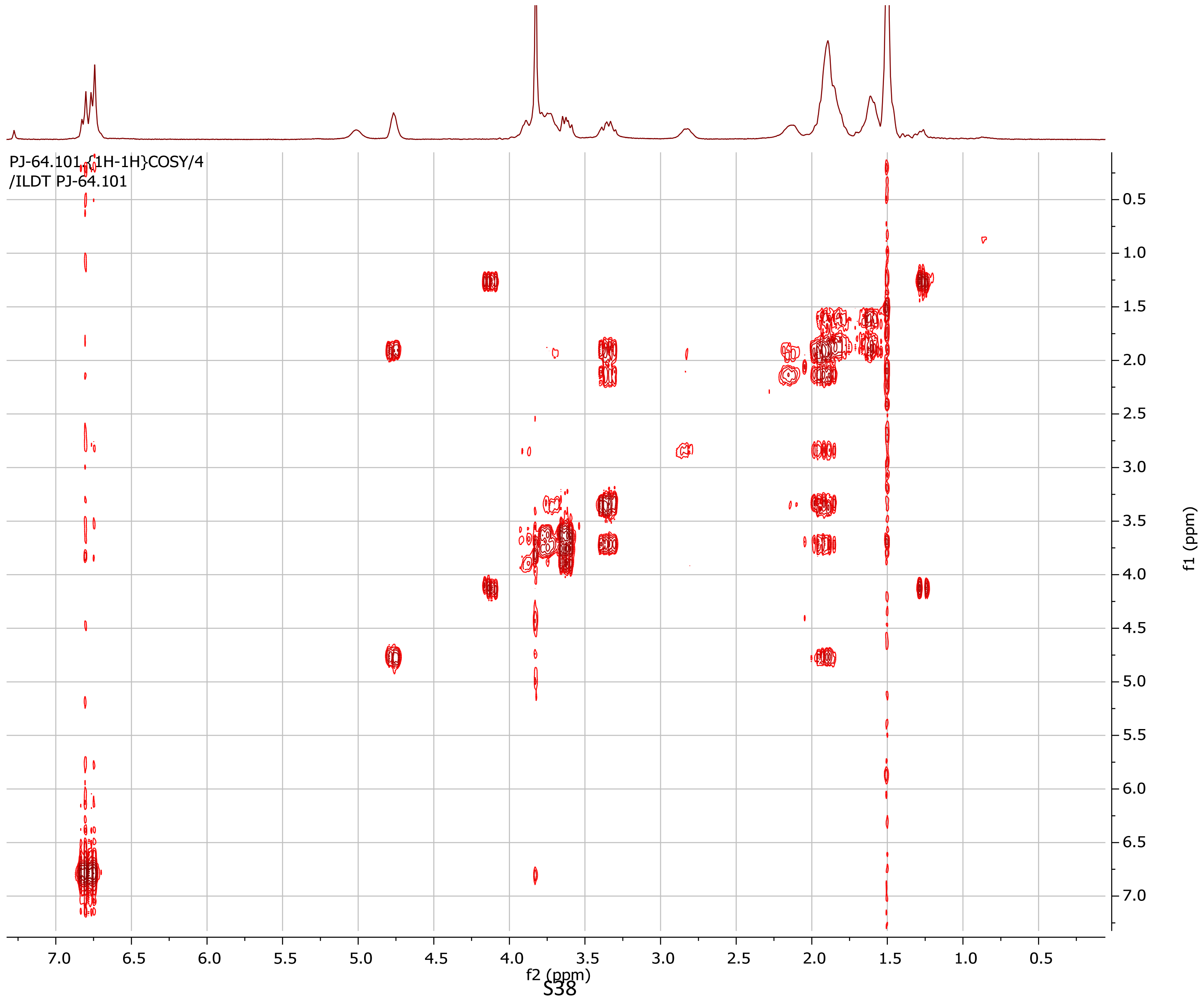
PJ-64.101.{1H-1H}COSY/4
/ILDT PJ-64.101
f2 (ppm)
f1 (ppm)

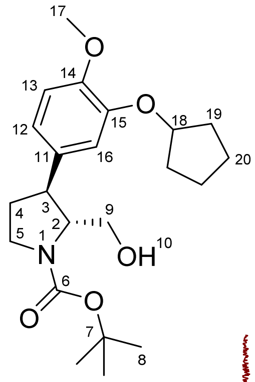

(-)-**20**

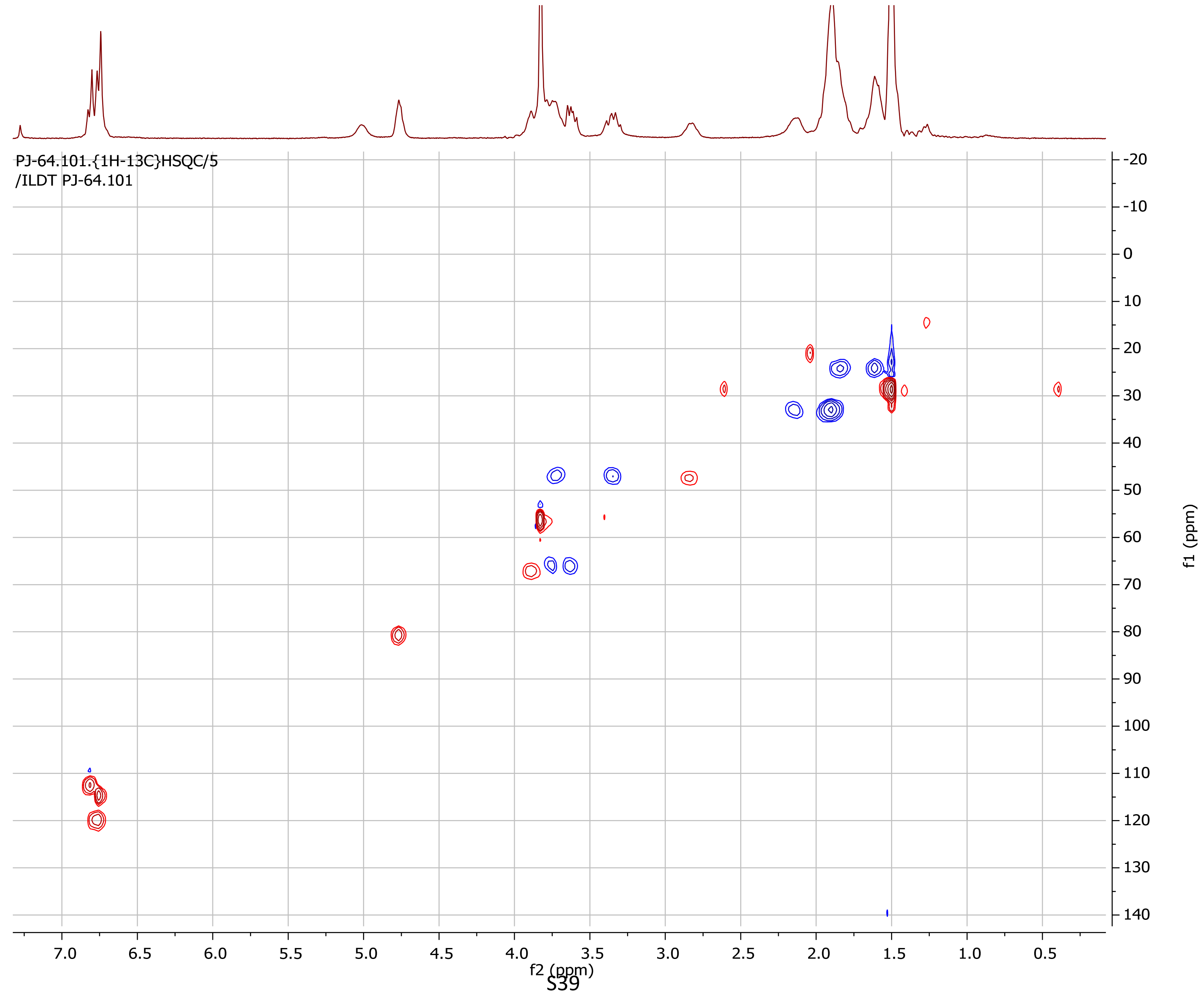

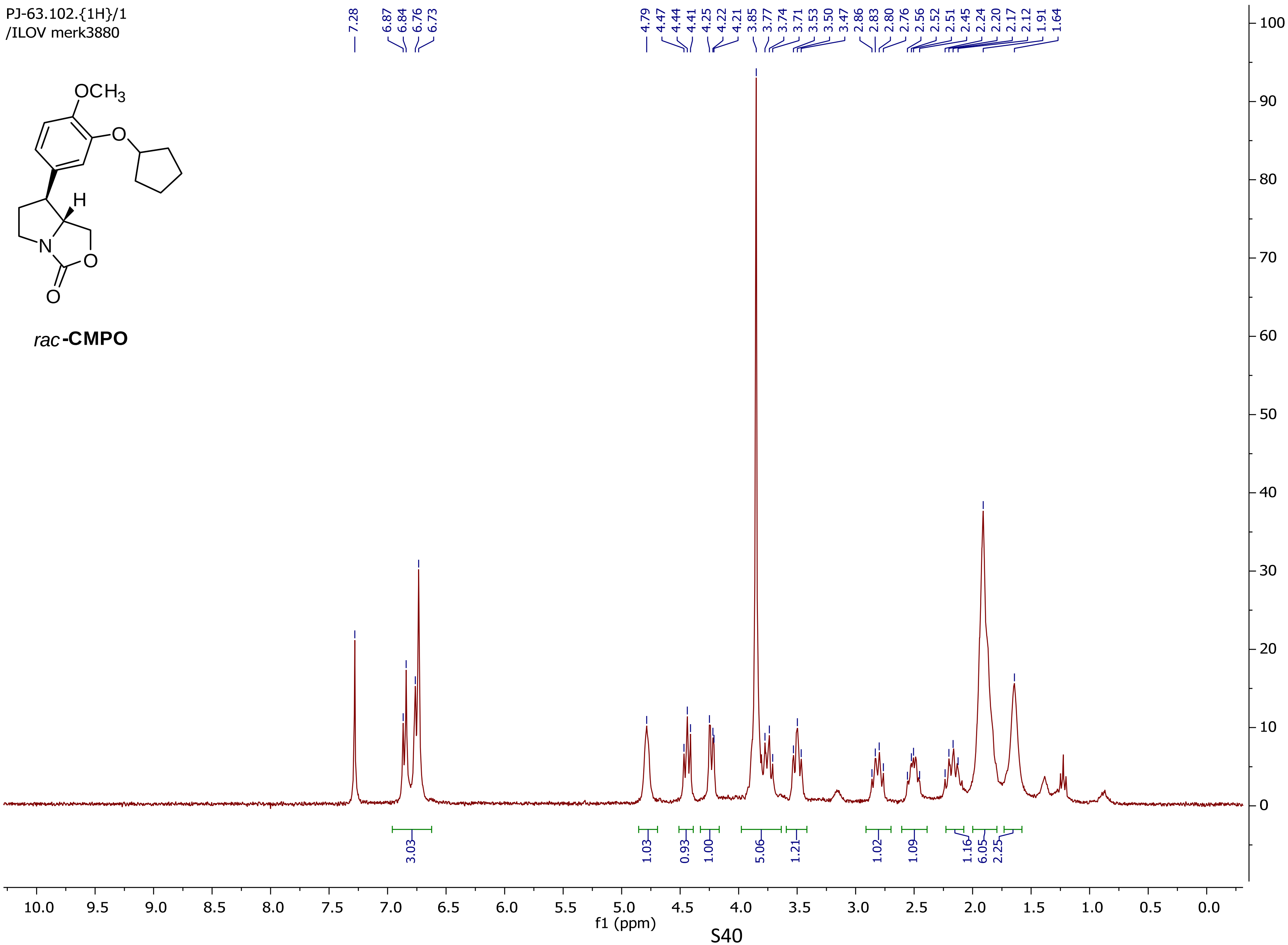
PJ-63.102.{1H}/1
/ILOV merk3880
7.28
6.87
6.84
6.76
6.73
4.79
4.47
4.44
4.41
4.25
4.22
4.21
3.85
3.77
3.74
3.71
3.53
3.50
3.47
2.86
2.83
2.80
2.76
2.56
2.52
2.51
2.45
2.24
2.20
2.17
2.12
1.91
1.64
OCH3
O
H
N
O
O
rac-CMPO
3.03
1.03
0.93
1.00
5.06
1.21
1.02
1.09
1.16
6.05
2.25
f1 (ppm)

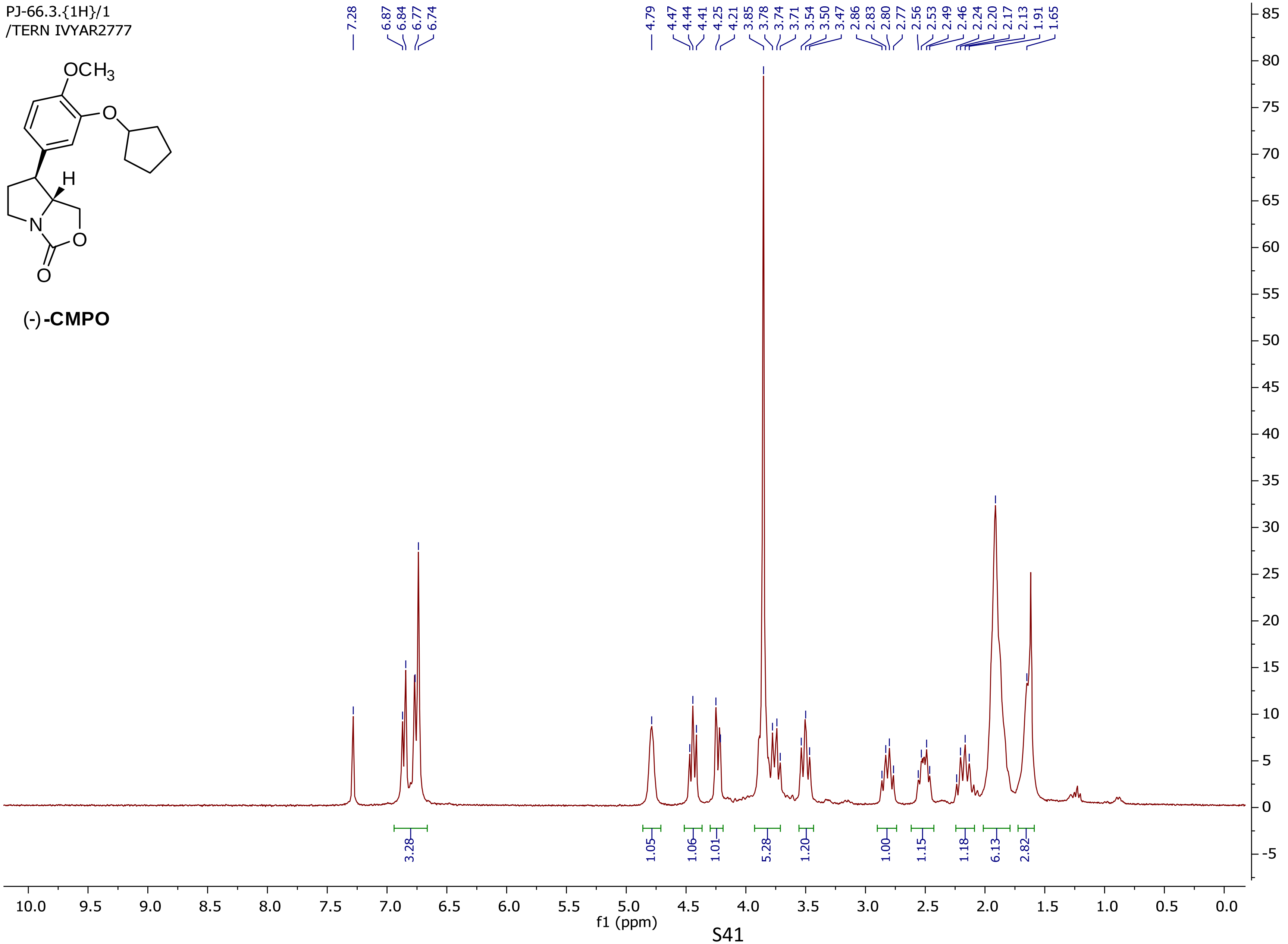
PJ-66.3.{1H}/1
/TERN IVYAR2777
OCH3
O
H
N
O
O
(-)-CMPO
7.28
6.87
6.84
6.77
6.74
4.79
4.47
4.44
4.41
4.25
4.21
3.85
3.78
3.74
3.71
3.54
3.50
3.47
2.86
2.83
2.80
2.77
2.56
2.53
2.49
2.46
2.24
2.20
2.17
2.13
1.91
1.65
3.28
1.05
1.06
1.01
5.28
1.20
1.00
1.15
1.18
6.13
2.82
f1 (ppm)

Analysis Report

SHIMADZU LabSolutions

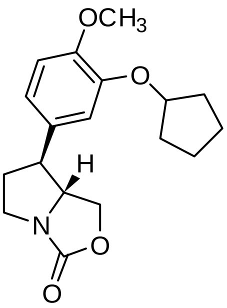

(-)-**CMPO**

<Sample Information>

Sample Name	: PJ066_chir		
Sample ID	: PJ066_chir		
Data Filename	: PJ066_chir.lcd		
Method Filename	: multiwave.lcm		
Batch Filename	:		
Vial #	: 1-7	Sample Type	: Unknown
Injection Volume	: 5 uL		
Date Acquired	: 10.09.2019 19:15:34	Acquired by	: PZ
Date Processed	: 24.03.2020 18:13:20	Processed by	: PZ

<Chromatogram>

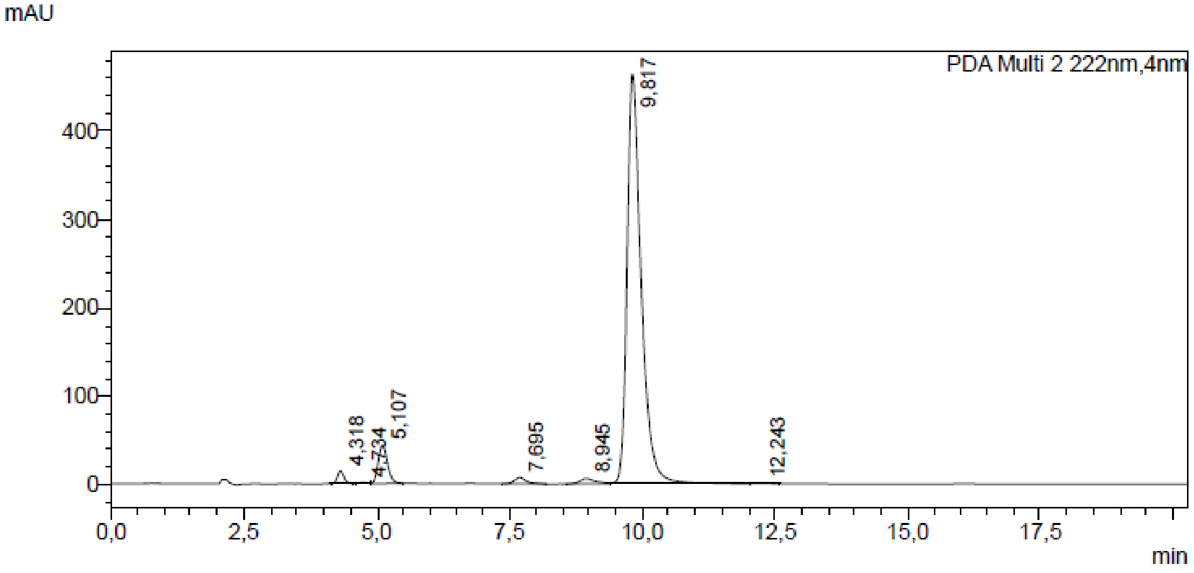

<Peak Table>

PDA Ch2 222nm

Peak#	Ret. Time	Area	Height	Conc.	Unit	Mark	Name
1	4,318	120304	14262	0,000			
2	4,734	14093	1156	0,000			
3	5,107	528612	43417	0,000		V	
4	7,695	104729	6981	0,000			
5	8,945	121911	5804	0,000			
6	9,817	8642800	465415	0,000		SV	
7	12,243	6411	351	0,000		TV	
Total		9538859	537386				

SHIMADZU LabSolutions

Analysis Report

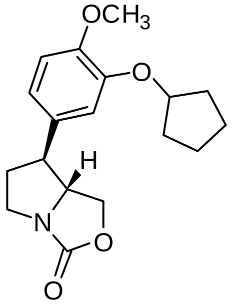

rac-**CMPO**

<Sample Information>

Sample Name	: PJ066_rac1		
Sample ID	: PJ066_rac1		
Data Filename	: PJ066_rac1.lcd		
Method Filename	: multiwave.lcm		
Batch Filename	:		
Vial #	: 1-6	Sample Type	: Unknown
Injection Volume	: 5 uL		
Date Acquired	: 10.09.2019 19:37:41	Acquired by	: PZ
Date Processed	: 10.09.2019 19:58:27	Processed by	: PZ

<Chromatogram>

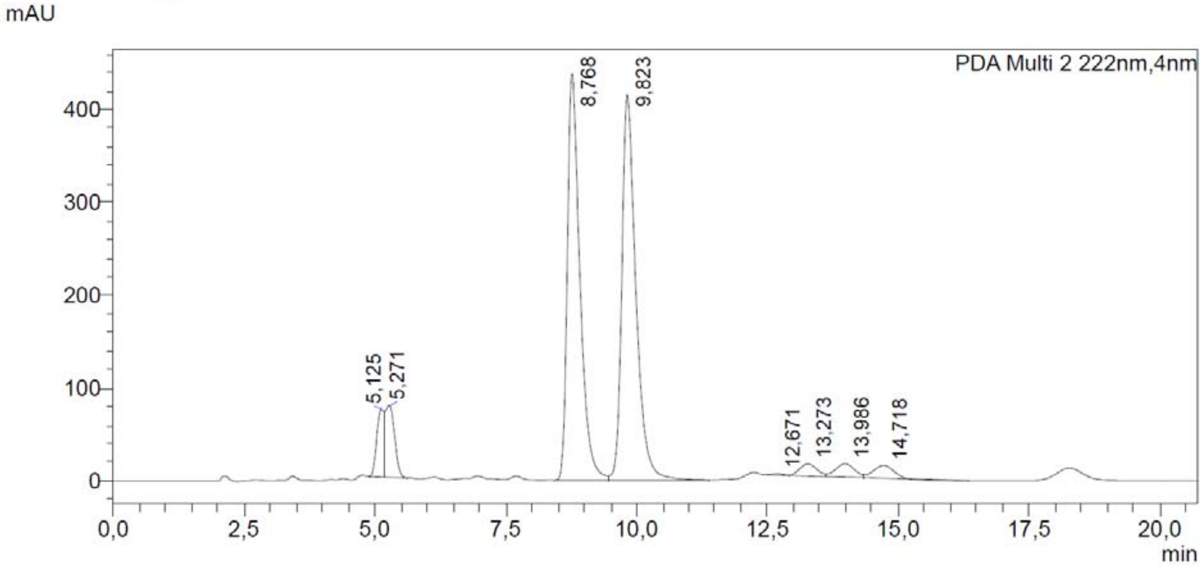

<Peak Table>

PDA Ch2 222nm

Peak#	Ret. Time	Area	Height	Conc.	Unit	Mark	Name
1	5,125	719699	72912	4,073			
2	5,271	937649	77751	5,307		V	
3	8,768	7266820	437934	41,126			
4	9,823	7719721	415104	43,690		V	
5	12,671	22999	1607	0,130			
6	13,273	302739	13099	1,713		V	
7	13,986	369097	14441	2,089		V	
8	14,718	330749	13775	1,872		SV	
Total		17669474	1046622				

DFT calculations

DFT calculations were performed with the Gaussian 16 Rev A.03 program.[1] Initially, preliminary calculations for several conformations of each compound were performed to define conformer with the lowest total energy. Then, DFT MN15/Def2TZVP level of theory was used for more precise geometry optimization and calculations of thermodynamics. All calculations were performed in acetic acid (SMD model), the approach of Martin and co-workers was followed.[2] Cartesian coordinates are given in angstroms, absolute energies for all substances are given in hartrees. Analysis of vibrational frequencies was performed for all optimized structures. All compounds were characterized by only real vibrational frequencies. Wavefunction stability, using *stable* keyword,[3] was also checked for each molecule.

For calculations of optimized geometries, frequencies and thermodynamics, following keywords were used:

# opt freq MN15/Def2TZVP SCF=YQC nosymm scrf=(smd,solvent=AceticAcid) pressure=428 temperature=298.15 test

Results

Table S1. Calculated relative free energies of cations **C2 – C4**

**Compound**	**$\Delta G°_{298,15\ K}$ Kcal/mol**
**C2**	0
**C3**	+13.2
**C4**	-7.5

Table S2. Calculated relative free energies of compounds **18** and **18'**

Compound	$\Delta G°_{298,15\ K}$ Kcal/mol
**18'**	0
**18**	+5.0

Cation C2

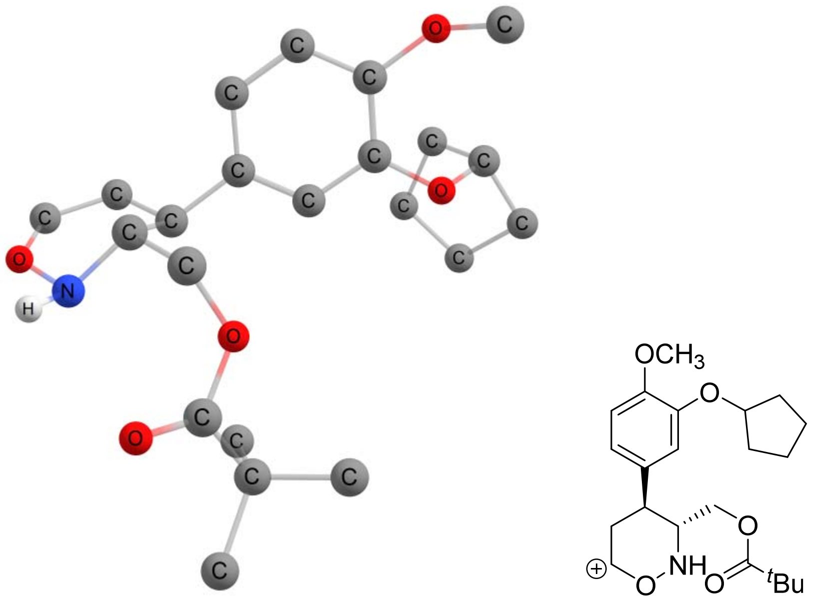

(hydrogens attached to carbon atoms are omitted for clarity)

O	-0.59676900	-2.83248900	-1.03439000
N	-0.29803200	-1.81738800	-1.97397900
C	-0.75693000	-0.51835500	-1.49167500
C	-2.28547800	-0.59229000	-1.42500500
H	-2.63032800	-0.88593500	-2.41907500
C	-2.63618000	-1.69907100	-0.43415000
H	-2.72618200	-1.33677800	0.60064300
H	-3.60605300	-2.17207100	-0.63202400
C	-1.65829900	-2.77726600	-0.36603400
H	-1.78852600	-3.63748000	0.28471500
C	-0.24396700	0.55414700	-2.43359100
H	0.83830300	0.49173800	-2.54907100
H	-0.50507000	1.52852900	-2.02413400
O	-0.88562100	0.45947600	-3.69817600
C	-0.29006800	-0.26867300	-4.65559100
O	0.79598000	-0.78052500	-4.49993400
H	0.71642600	-1.88930800	-2.06790200
H	-0.35108600	-0.31082700	-0.49086300
C	-2.89654100	0.73978000	-1.07291500
C	-3.61072400	1.44104800	-2.03755400
C	-2.70772600	1.31276800	0.18280900
C	-4.13994900	2.70017000	-1.77205000
H	-3.77141600	1.01754900	-3.02521100
C	-3.22450000	2.57149200	0.45543400
H	-2.14599000	0.79022900	0.94992500
C	-3.92951100	3.27376300	-0.51245100
H	-3.08362900	3.03918800	1.42258600
C	-1.15536400	-0.38567800	-5.89053600
C	-2.40436300	-1.17931300	-5.49095500
C	-1.56173300	1.00466800	-6.37850700
C	-0.38383900	-1.12743600	-6.97148700
H	-2.13510800	-2.14183800	-5.04664600
H	-3.01742300	-0.62055600	-4.77947500
H	-3.00495100	-1.37180900	-6.38276000
H	-0.68669500	1.60559100	-6.63567100
H	-2.17708700	0.89880600	-7.27474900
H	-2.14267800	1.53832300	-5.62437800

H	-1.00813600	-1.21406800	-7.86314200
H	0.52977600	-0.59394300	-7.24008000
H	-0.10817600	-2.13108600	-6.64310200
O	-4.43196700	4.51087900	-0.21297900
C	-3.72250000	5.58050400	-0.83035900
H	-3.67203000	5.44836100	-1.91452400
H	-4.25942300	6.49837200	-0.59946900
H	-2.70749100	5.64176100	-0.42832500
O	-4.79910400	3.33021400	-2.77874500
C	-6.08779400	3.94366700	-2.54810000
C	-6.76970000	3.94022700	-3.90299000
C	-6.98452600	3.03650200	-1.72628300
H	-5.95351700	4.93993400	-2.12509200
C	-7.18308100	2.47349000	-4.10328600
H	-6.11420900	4.32004500	-4.68771400
H	-7.64439500	4.59206800	-3.84680500
C	-7.28131000	1.87309800	-2.68027200
H	-7.89353800	3.59684600	-1.49454800
H	-6.53999400	2.72756300	-0.77844500
H	-8.12285000	2.39484000	-4.65012600
H	-6.42799400	1.94479900	-4.68721800
H	-8.25475900	1.42467900	-2.48136300
H	-6.53330000	1.08704400	-2.54891300

DFT MN15/Def2TZVP, solvent acetic acid, SMD model		
Sum of electronic and zero-point Energies=	-1286.693718	$E_0 + E_{ZPE}$
Sum of electronic and thermal Energies=	-1286.665915	$E_0 + E_{tot}$
Sum of electronic and thermal Enthalpies=	-1286.664970	$E_0 + H_{corr}$
Sum of electronic and thermal Free Energies=	-1286.746281	$E_0 + G_{corr}$
Zero-point correction *(unscaled)* =	0.518323	

Cation C3

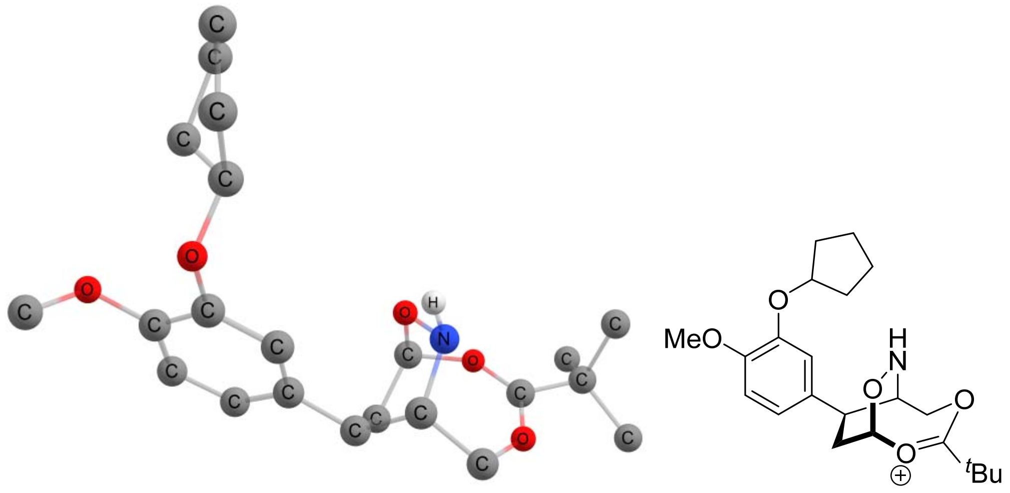

(hydrogens attached to carbon atoms are omitted for clarity)

C	-1.52042800	0.71703600	0.96474500
C	-1.75605200	-0.78705100	1.04369500
O	-2.48316800	-1.29129700	-0.00629500
N	-1.75826600	-1.13558700	-1.22658700
C	-1.34755800	0.26180100	-1.42133800
C	-1.97387700	1.21471600	-0.40762300
C	-4.15285900	2.16559200	0.40806900
C	-4.20622400	0.87306900	-1.59966600
C	-5.57390300	1.09408900	-1.71415800
C	0.17602100	0.32991100	-1.35761400
O	0.83913300	-0.62831300	-0.48640400
C	0.54241500	-1.48155900	0.40499500
O	-0.50587000	-1.55027000	1.13091300
C	1.55649800	-2.56754500	0.63600700
C	2.95990100	-2.09159100	0.27999400
C	1.49910600	-3.07650000	2.07145900
C	1.11537200	-3.67919900	-0.33507500
C	-3.47532100	1.39272200	-0.53580700
C	-5.51424100	2.40313300	0.29441300
C	-6.23935600	1.86435500	-0.76024900
H	-0.46599100	0.94813100	1.12927300
H	-2.07057600	1.19426500	1.77245600
H	-2.27687400	-1.08840100	1.94685000
H	-2.44292100	-1.42317800	-1.92125300
H	-1.61237200	0.56163300	-2.43696100
H	-1.51337800	2.19178000	-0.57751500
H	-3.61196100	2.61064700	1.23514400
H	-3.74328300	0.28716400	-2.38606300
H	0.61236400	0.09200200	-2.32431500
H	0.52993900	1.30884100	-1.03751200
H	3.65452900	-2.91337000	0.45914000
H	3.03514900	-1.80328400	-0.76829300
H	3.26170400	-1.24604700	0.90088800
H	2.24637200	-3.86313700	2.18534100
H	0.52264400	-3.49566900	2.31246500
H	1.72745500	-2.28131400	2.78351800
H	0.10653700	-4.02662700	-0.10354000
H	1.14580100	-3.33269800	-1.36997300
H	1.80797600	-4.51573000	-0.22647600
H	-6.03854200	3.00614000	1.02611700

O	-7.59002100	2.06315700	-0.82783100
C	-8.00562000	2.96425400	-1.84838900
H	-9.09219900	3.00912000	-1.81089000
H	-7.59532100	3.96120200	-1.66334000
H	-7.68955500	2.61683100	-2.83392000
O	-6.26232900	0.57198000	-2.77733400
C	-6.61170700	-0.80706400	-2.64516000
C	-7.73491500	-1.08228900	-1.65866800
C	-7.15957200	-1.33014900	-3.96470300
H	-5.71422100	-1.36539500	-2.34344100
C	-8.10934300	-2.51186600	-2.03147300
H	-8.55784300	-0.39431800	-1.88018600
H	-7.44391000	-0.95018200	-0.61505200
C	-8.05788300	-2.52181100	-3.57327100
H	-6.36995400	-1.59182300	-4.66906400
H	-7.74917300	-0.52601800	-4.41249200
H	-7.36206700	-3.20000000	-1.62626400
H	-9.07841600	-2.81765800	-1.63768400
H	-7.67269100	-3.46800500	-3.95376400
H	-9.05726800	-2.39578800	-3.99126000

DFT MN15/Def2TZVP, solvent acetic acid, SMD model		
Sum of electronic and zero-point Energies=	-1286.671690	$E_0 + E_{ZPE}$
Sum of electronic and thermal Energies=	-1286.644417	$E_0 + E_{tot}$
Sum of electronic and thermal Enthalpies=	-1286.643473	$E_0 + H_{corr}$
Sum of electronic and thermal Free Energies=	-1286.725249	$E_0 + G_{corr}$
Zero-point correction *(unscaled)* =	0.521611	

Cation C4

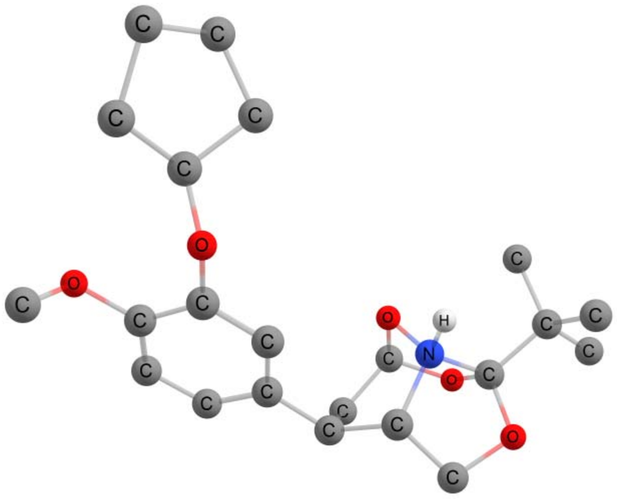

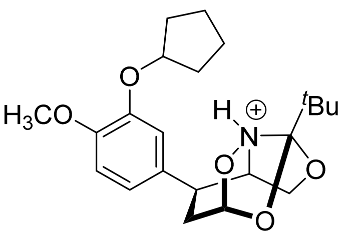

(hydrogens attached to carbon atoms are omitted for clarity)

C	-1.73805400	0.83545900	1.62888400
C	-1.34951800	-0.63062800	1.60476300
O	-1.77678500	-1.19509900	0.36163900
N	-0.77051900	-0.75902300	-0.48582300
C	-0.75314300	0.75257200	-0.67373300
C	-1.83413200	1.38947000	0.18822200
C	-4.25138200	2.02720600	0.23780000
C	-3.56208800	0.53999800	-1.50370300
C	-4.86154700	0.49816400	-1.99544600
C	0.66171000	1.13654900	-0.21306500
O	1.37747700	-0.08974300	-0.14849800
C	0.53056700	-1.05765900	0.29056500
O	0.05496300	-0.82479600	1.58393500
C	1.10266500	-2.45555500	0.08847100
C	1.57089800	-2.62375100	-1.35370800
C	2.29217100	-2.58278400	1.04044200
C	0.06057400	-3.51753900	0.42616700
C	-3.23716700	1.30074900	-0.38613600
C	-5.54947600	2.00018500	-0.25015100
C	-5.86402200	1.24362500	-1.37038200
H	-1.00397400	1.37380600	2.22855100
H	-2.70326100	0.93850100	2.12249100
H	-1.78756700	-1.22915000	2.39884100
H	-0.85856600	-1.29040000	-1.36153900
H	-0.89285200	0.94175700	-1.73455200
H	-1.56679400	2.44968700	0.22790600
H	-4.01962200	2.63207800	1.10830300
H	-2.83098700	-0.05557300	-2.03935800
H	1.16311700	1.78364000	-0.92596700
H	0.64200100	1.61392300	0.76783300
H	1.94905100	-3.63951600	-1.48111300
H	0.75655100	-2.49182800	-2.07424900
H	2.37169900	-1.92679000	-1.60082600
H	2.74529900	-3.56603500	0.90216300
H	1.97580400	-2.49428800	2.08102600
H	3.04803400	-1.82323600	0.83447800
H	-0.34425300	-3.38247900	1.43102900
H	-0.76657100	-3.53206300	-0.28755800
H	0.54648900	-4.49398500	0.38689300
O	-7.14732400	1.21637600	-1.84669500
C	-7.33414300	1.98583500	-3.03124600

H	-8.36928700	1.85528200	-3.34025400
H	-6.66881500	1.64801200	-3.82950000
O	-5.12481500	-0.24385200	-3.10703300
H	-6.33762400	2.57339400	0.22328700
H	-7.14781700	3.04378900	-2.82606300
C	-6.05863300	-1.32565500	-2.96148700
C	-7.11792800	-1.30733300	-4.06830800
C	-5.37720400	-2.67831300	-3.09803800
H	-6.52695500	-1.24561400	-1.97686000
C	-7.34568000	-2.78517000	-4.44712800
H	-6.72579100	-0.73355500	-4.91094000
H	-8.02889500	-0.81145500	-3.73148100
C	-6.55714900	-3.58968800	-3.40520700
H	-4.80448300	-2.95828600	-2.21203500
H	-4.69934300	-2.63672500	-3.95794700
H	-8.40122500	-3.05530300	-4.47127900
H	-6.93957500	-2.98345700	-5.44123500
H	-7.15740600	-3.72786900	-2.50075000
H	-6.26323700	-4.57741700	-3.76106100

DFT MN15/Def2TZVP, solvent acetic acid, SMD model		
Sum of electronic and zero-point Energies=	-1286.707606	$E_0 + E_{ZPE}$
Sum of electronic and thermal Energies=	-1286.681915	$E_0 + E_{tot}$
Sum of electronic and thermal Enthalpies=	-1286.680970	$E_0 + H_{corr}$
Sum of electronic and thermal Free Energies=	-1286.758293	$E_0 + G_{corr}$
Zero-point correction *(unscaled)* =	0.524286	

Compound 18’

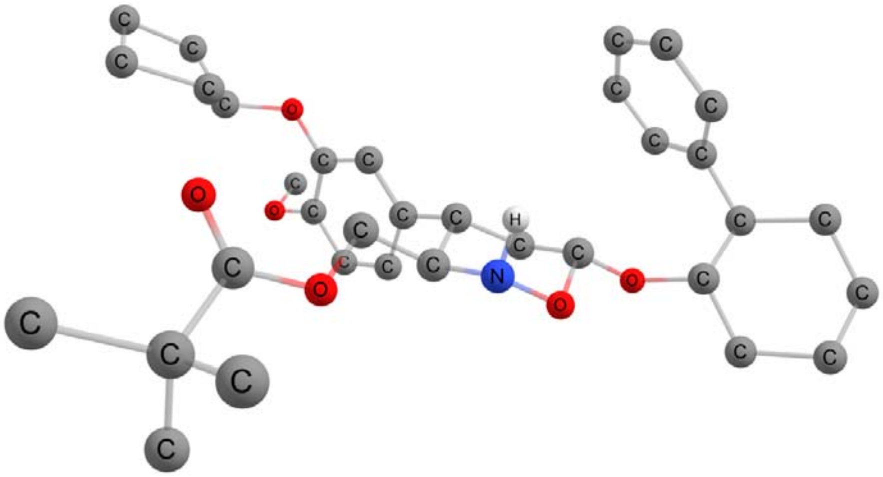

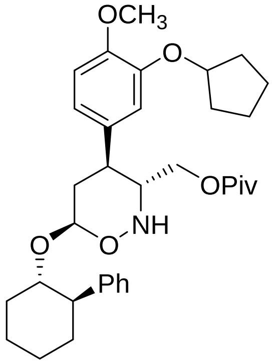

(hydrogens attached to carbon atoms are omitted for clarity)

C	0.21441900	-2.00864100	-1.10280800
C	-0.84803000	-0.93169000	-1.37636100
N	-1.15883500	-0.14544800	-0.18620900
O	-1.58502200	-0.99102300	0.85634400
C	-0.54141800	-1.88493700	1.24806300
C	-0.37179700	0.01374700	-2.45370400
O	-1.00664700	-2.59877000	2.33428800
C	-0.21886000	-2.82253400	0.11336100
O	-1.43643900	0.90160400	-2.79287800
C	-1.19582800	1.75749200	-3.78652100
C	-2.37796300	2.66209600	-4.06773600
O	-0.13351200	1.78224700	-4.37350100
C	-2.69049600	3.46150700	-2.80081400
C	-3.58329800	1.79679600	-4.44085000
C	-2.02771600	3.60117000	-5.21176100
H	-0.30467600	0.32075000	0.14226800
H	0.48941000	0.59539600	-2.10713600
H	-0.07025000	-0.54125900	-3.34345300
H	0.57196700	-3.51095900	0.41936500
H	-1.12184700	-3.40100500	-0.10265000
H	-3.52953000	4.13218200	-3.00104300
H	-1.83432500	4.06807000	-2.49580800
H	-2.96339900	2.80301200	-1.97476500
H	-4.42989300	2.44738700	-4.67286300
H	-3.37279300	1.18407900	-5.32064800
H	-3.86889600	1.14030200	-3.61782500
H	-1.16598100	4.22330200	-4.96292500
H	-2.87899200	4.25484800	-5.41463200
H	-1.79382200	3.04578000	-6.12192200
H	-1.77655500	-1.41564100	-1.69877000
H	1.14535500	-1.48167700	-0.84699200
C	0.46991700	-2.84978100	-2.32782700
C	1.64351700	-2.67661100	-3.05741200
C	-0.49107200	-3.73723600	-2.81004800
C	1.85762300	-3.36013600	-4.24510400
H	2.40279600	-1.97593400	-2.72337600
C	-0.27447300	-4.44258300	-3.98728900
H	-1.42061100	-3.87842800	-2.26983700
C	0.89498500	-4.25915000	-4.71123100
H	-1.01313100	-5.13811100	-4.36800400
O	1.08383700	-4.92743000	-5.89296000

C	2.02941400	-5.98823800	-5.81072700
H	3.00056000	-5.62300800	-5.46968000
H	2.12996300	-6.40629000	-6.81048000
H	1.67050500	-6.76344900	-5.12760500
O	3.00809200	-3.14937800	-4.95857800
C	2.83935500	-2.27981800	-6.08353800
C	4.13024000	-2.23991800	-6.88985000
C	2.59350100	-0.82316300	-5.71776700
H	2.00667700	-2.65333700	-6.69149600
C	4.12675900	-0.86236800	-7.58495600
H	4.95883500	-2.31961700	-6.18136800
H	4.20790800	-3.07745200	-7.58325000
C	2.88074400	-0.13327400	-7.04631500
H	1.59327200	-0.62478100	-5.32639400
H	3.33045800	-0.52828400	-4.96147400
H	4.10227600	-0.94930000	-8.67137600
H	5.03197500	-0.30880700	-7.33110700
H	2.03431400	-0.28859500	-7.72102200
H	3.02429600	0.94264500	-6.94830900
C	-1.07377100	-1.88127300	3.57004100
C	0.09407400	-2.29365700	4.47316900
C	-2.41186300	-2.18647800	4.21203800
H	-0.99707100	-0.80390600	3.36364200
C	-0.04771800	-1.60465500	5.83007600
H	0.00254600	-3.37799200	4.62009100
C	-2.53256100	-1.53003300	5.58039500
H	-2.49283200	-3.27531500	4.31267000
H	-3.20685600	-1.85898200	3.53746500
C	-1.37948400	-1.95352200	6.48090600
H	0.79280900	-1.88460200	6.47031000
H	-0.00001600	-0.51776500	5.69670200
H	-3.49338500	-1.78402600	6.03329000
H	-2.51357400	-0.43992800	5.46328000
H	-1.45944700	-1.47161200	7.45816800
H	-1.42906700	-3.03567700	6.65028000
C	1.39691200	-2.02107200	3.76453000
C	2.01973900	-3.03808900	3.04274800
C	1.93525800	-0.73476000	3.69793400
C	3.13468200	-2.77859000	2.25377900
H	1.60491600	-4.04009100	3.08288800
C	3.05283800	-0.47044400	2.91500300
H	1.46541300	0.07794600	4.24080800
C	3.65195000	-1.48994300	2.18220900
H	3.59795500	-3.58197300	1.69314700
H	3.45349500	0.53547000	2.87193600
H	4.51986800	-1.28288200	1.56794200
H	0.33455600	-1.26709900	1.51186800

DFT MN15/Def2TZVP, solvent acetic acid, SMD model		
Sum of electronic and zero-point Energies=	-1827.746933	$E_0 + E_{ZPE}$
Sum of electronic and thermal Energies=	-1827.706055	$E_0 + E_{tot}$
Sum of electronic and thermal Enthalpies=	-1827.705110	$E_0 + H_{corr}$
Sum of electronic and thermal Free Energies=	-1827.822596	$E_0 + G_{corr}$
Zero-point correction *(unscaled)* =	0.763610	

Compound 18

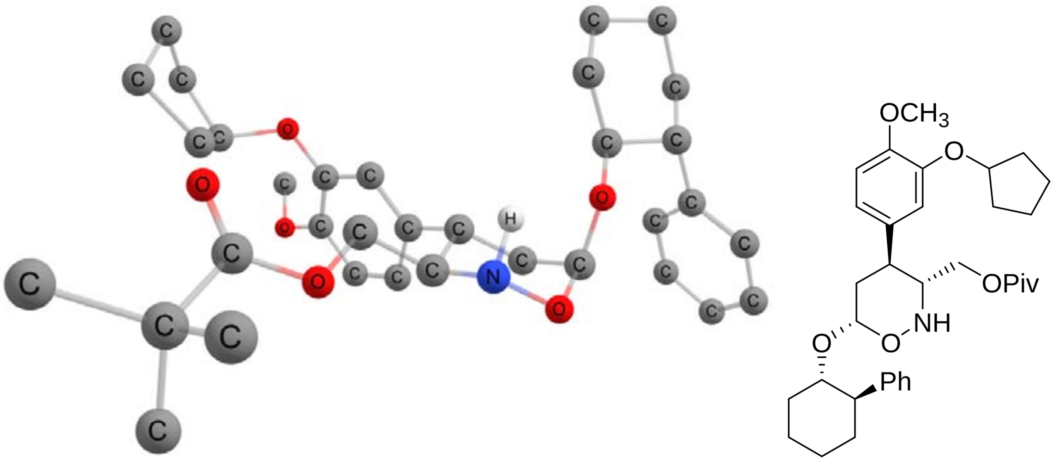

C	0.23055900	-1.87192600	-1.01483700
C	-0.83119500	-0.83143700	-1.41858600
N	-1.15952100	0.06757500	-0.31814500
O	-1.60022500	-0.66120300	0.80972700
C	-0.61166600	-1.53862400	1.30901500
C	-0.33732100	0.00527500	-2.57599800
O	0.45462600	-0.70493100	1.70147800
C	-0.26647200	-2.56585900	0.24651400
O	-1.37210100	0.89807300	-2.98739500
C	-1.09469800	1.67057200	-4.03837400
C	-2.24622400	2.58487700	-4.40329000
O	-0.02449900	1.62354700	-4.61012900
C	-2.52820200	3.50492700	-3.21313400
C	-3.48191600	1.73296700	-4.69912800
C	-1.86513500	3.40507100	-5.62643400
H	-0.30784800	0.55317700	-0.01814900
H	0.54704300	0.58389000	-2.28481300
H	-0.06562900	-0.63211200	-3.41784900
H	0.45206400	-3.30112600	0.61149700
H	-1.19758600	-3.10256200	0.04477500
H	-3.34664300	4.18108500	-3.47170800
H	-1.65205300	4.10877500	-2.96493100
H	-2.81820900	2.93236800	-2.33072300
H	-4.30485300	2.38955300	-4.99151500
H	-3.29441100	1.03549500	-5.51905800
H	-3.79046900	1.16474000	-3.82068500
H	-0.98259000	4.01737100	-5.43242200
H	-2.69401400	4.06632800	-5.88883900
H	-1.65087200	2.76237600	-6.48247000
H	-1.75001200	-1.35465600	-1.70827300
H	1.14546200	-1.31332900	-0.77360400
C	0.54771400	-2.80938800	-2.15208700
C	1.64740000	-2.54449600	-2.96370900
C	-0.28947300	-3.86671500	-2.50452100
C	1.90209100	-3.27764300	-4.11400000
H	2.30436000	-1.70886900	-2.74030700
C	-0.01046200	-4.64667600	-3.61910300
H	-1.16618900	-4.09463300	-1.90947400
C	1.08461200	-4.37011300	-4.42875100
H	-0.64093200	-5.48681600	-3.88525300
O	1.28896800	-5.15572600	-5.52489700
C	2.61349300	-5.61333500	-5.77687300

H	3.14536100	-4.94771900	-6.45972800
H	2.52369300	-6.59774300	-6.23424300
H	3.17751200	-5.69733600	-4.84575200
O	2.93178400	-2.90121400	-4.93406600
C	2.48941300	-2.32480500	-6.18411500
C	3.73579400	-2.01453100	-7.00795500
C	1.79513000	-0.96710500	-5.95633400
H	1.83535500	-3.05050400	-6.67411500
C	4.01874700	-0.54335800	-6.71675800
H	4.54956600	-2.69891500	-6.76220700
H	3.49909800	-2.13421800	-8.06873500
C	2.61478700	0.05197900	-6.75376400
H	0.73683400	-0.97801100	-6.21952800
H	1.86150600	-0.72423400	-4.89242300
H	4.71039100	-0.08717300	-7.42592900
H	4.44088900	-0.43690300	-5.71198300
H	2.26715100	0.09454500	-7.79057200
H	2.54324000	1.05737300	-6.33660300
H	-1.08495300	-2.01296800	2.17481500
C	1.76063500	-1.21155500	1.94643700
C	1.95061700	-1.54696200	3.42822000
C	2.74619800	-0.14304200	1.50914300
H	1.93604200	-2.12212200	1.35823800
C	3.39064400	-2.00814200	3.66241200
H	1.78883600	-0.61264300	3.98149400
C	4.18121900	-0.58639000	1.76093800
H	2.52878900	0.76873100	2.07778000
H	2.58247400	0.08485800	0.45112500
C	4.38412400	-0.93905000	3.22859000
H	3.52251100	-2.26249300	4.71711300
H	3.57352700	-2.92483300	3.08825900
H	4.87533200	0.19786100	1.45133400
H	4.40088800	-1.46751400	1.14609700
H	5.40739900	-1.28000700	3.40258300
H	4.23585500	-0.04084900	3.83955400
C	0.92184300	-2.54874100	3.88347100
C	-0.09582000	-2.17160300	4.75695700
C	0.91995500	-3.85013900	3.37926100
C	-1.10332600	-3.06352900	5.10733200
H	-0.10681700	-1.15974300	5.14827600
C	-0.08600600	-4.74447900	3.72318300
H	1.70343100	-4.16271800	2.69587700
C	-1.10493100	-4.35167700	4.58541500
H	-1.89117000	-2.74971500	5.78164300
H	-0.07701200	-5.74888800	3.31665800
H	-1.89225500	-5.04675200	4.85058500

DFT MN15/Def2TZVP, solvent acetic acid, SMD model		
Sum of electronic and zero-point Energies=	-1827.743648	$E_0 + E_{ZPE}$
Sum of electronic and thermal Energies=	-1827.703710	$E_0 + E_{tot}$
Sum of electronic and thermal Enthalpies=	-1827.702765	$E_0 + H_{corr}$
Sum of electronic and thermal Free Energies=	-1827.814669	$E_0 + G_{corr}$
Zero-point correction *(unscaled)* =	0.765533	

References

1. Gaussian 16, Revision A.03, M. J. Frisch, G. W. Trucks, H. B. Schlegel, G. E. Scuseria, M. A. Robb, J. R. Cheeseman, G. Scalmani, V. Barone, G. A. Petersson, H. Nakatsuji, X. Li, M. Caricato, A. V. Marenich, J. Bloino, B. G. Janesko, R. Gomperts, B. Mennucci, H. P. Hratchian, J. V. Ortiz, A. F. Izmaylov, J. L. Sonnenberg, D. Williams-Young, F. Ding, F. Lipparini, F. Egidi, J. Goings, B. Peng, A. Petrone, T. Henderson, D. Ranasinghe, V. G. Zakrzewski, J. Gao, N. Rega, G. Zheng, W. Liang, M. Hada, M. Ehara, K. Toyota, R. Fukuda, J. Hasegawa, M. Ishida, T. Nakajima, Y. Honda, O. Kitao, H. Nakai, T. Vreven, K. Throssell, J. A. Montgomery, Jr., J. E. Peralta, F. Ogliaro, M. J. Bearpark, J. J. Heyd, E. N. Brothers, K. N. Kudin, V. N. Staroverov, T. A. Keith, R. Kobayashi, J. Normand, K. Raghavachari, A. P. Rendell, J. C. Burant, S. S. Iyengar, J. Tomasi, M. Cossi, J. M. Millam, M. Klene, C. Adamo, R. Cammi, J. W. Ochterski, R. L. Martin, K. Morokuma, O. Farkas, J. B. Foresman, and D. J. Fox, Gaussian, Inc., Wallingford CT, **2016**.

2. Martin, R. L.; Hay, P. J.; Pratt, L. R, *J. Phys. Chem. A*, **1998**, 102, 3565.

3. For detailed description of all keywords, basis sets and functionals please refer to Gaussian 16 manual or http://www.gaussian.com/keywords and references therein.
